# Supplementary material for: Humans decompose tasks by trading off utility and computational cost
Source: PLoS Comput Biol. 2023 Jun 1;19(6):e1011087. doi: 10.1371/journal.pcbi.1011087 (PMC10234566; doi:10.1371/journal.pcbi.1011087)
Supplement: S1 Appendix — Extended data and analyses. (PDF) [file pcbi.1011087.s001.pdf]

Appendix

Extended Data

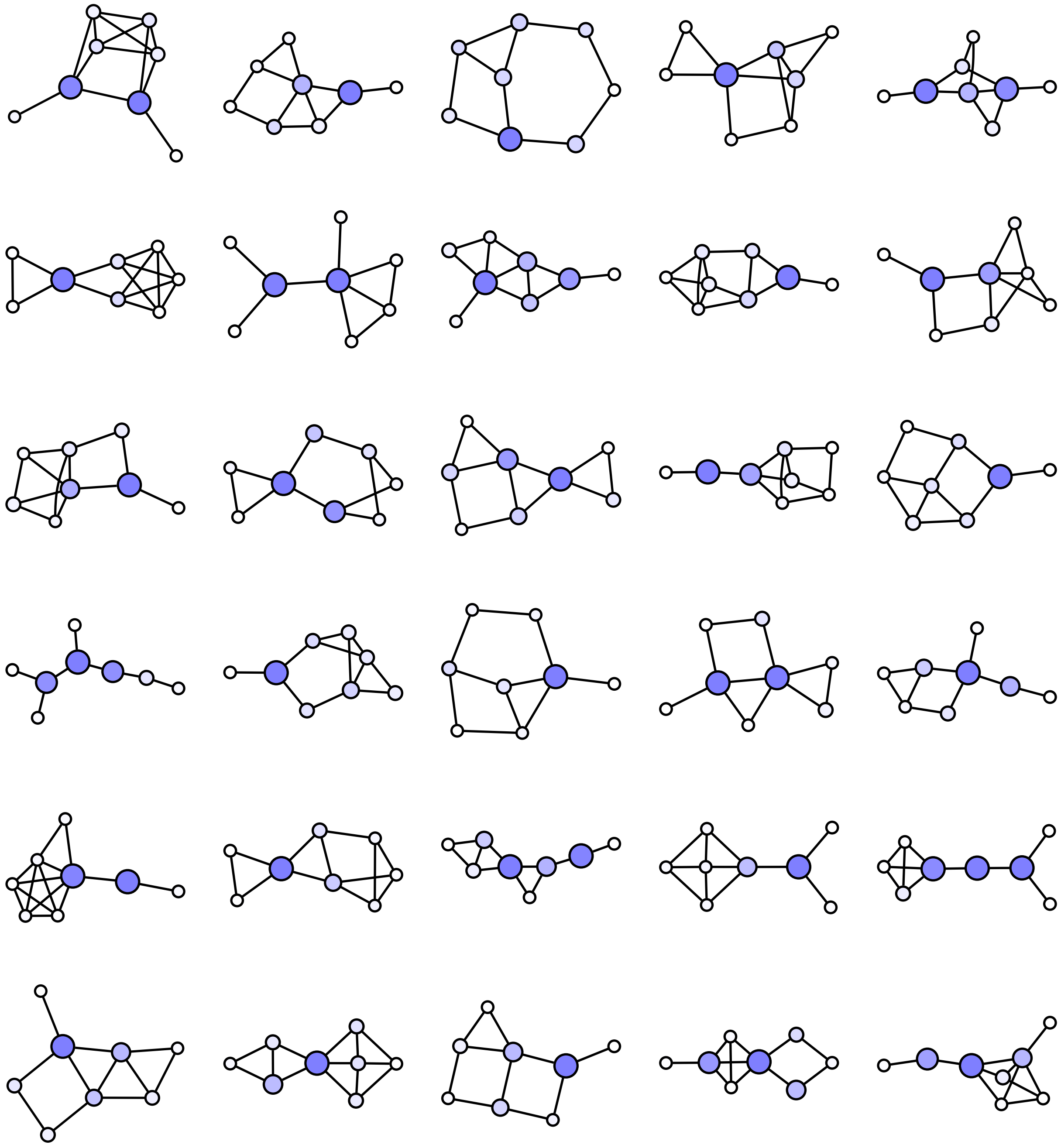

Fig A1. Visualization of participant behavior by graph. State color and size is proportional to subgoal choice, summed across participants and probe types. Participants per graph ranged from 21–30 and each participant responded to a total of 21 probe questions.

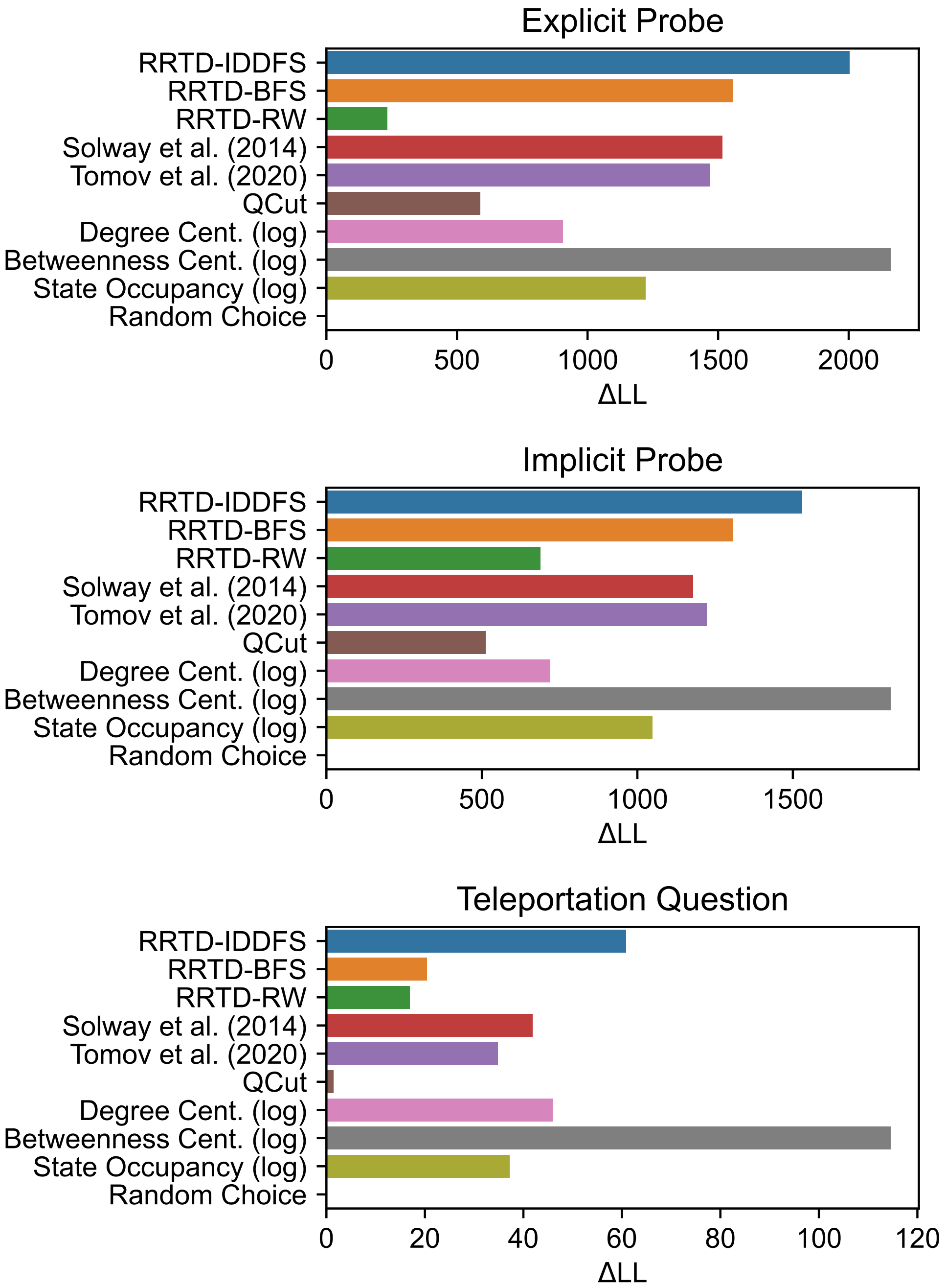

Fig A2. Comparison of probe choice behavior in the subset of participants ($N = 603$) that reported not using an additional visual aid during the experiment. The analysis is otherwise identical to that in the main text, using mixed-effects multinomial regression to predict subgoal choice behavior for each subgoal probe. Log likelihood (LL) is relative to the minimum model LL for each probe. Larger values indicate better predictivity.

Table A1. Estimated coefficients with standard errors from hierarchical multinomial regression predicting subgoal choice, in the subset of participants ($N = 603$) that reported no use of visual aid in the experiment. Likelihood-ratio test statistics compare regression models to the null hypothesis of sampling subgoals uniformly at random.

Normative Algorithm	Explicit Probe	Implicit Probe	Teleportation Question
RRTD-IDDFS	$\beta = 1.84$<br>$SE = 0.04$<br>$\chi^2(2) = 4004.7$<br>$p < .001$	$\beta = 1.69$<br>$SE = 0.04$<br>$\chi^2(2) = 3058.4$<br>$p < .001$	$\beta = 0.76$<br>$SE = 0.07$<br>$\chi^2(1) = 121.6$<br>$p < .001$
RRTD-BFS	$\beta = 4.47$<br>$SE = 0.11$<br>$\chi^2(2) = 3114.7$<br>$p < .001$	$\beta = 5.25$<br>$SE = 0.14$<br>$\chi^2(2) = 2614.5$<br>$p < .001$	$\beta = 1.51$<br>$SE = 0.24$<br>$\chi^2(1) = 40.8$<br>$p < .001$
RRTD-RW	$\beta = 0.35$<br>$SE = 0.03$<br>$\chi^2(2) = 467.7$<br>$p < .001$	$\beta = 0.93$<br>$SE = 0.03$<br>$\chi^2(2) = 1375.0$<br>$p < .001$	$\beta = 0.31$<br>$SE = 0.06$<br>$\chi^2(1) = 33.9$<br>$p < .001$
Solway et al. (2014) [1]	$\beta = 0.76$<br>$SE = 0.02$<br>$\chi^2(2) = 3031.7$<br>$p < .001$	$\beta = 0.70$<br>$SE = 0.02$<br>$\chi^2(2) = 2357.7$<br>$p < .001$	$\beta = 0.37$<br>$SE = 0.04$<br>$\chi^2(1) = 83.7$<br>$p < .001$
Tomov et al. (2020) [2]	$\beta = 1.13$<br>$SE = 0.03$<br>$\chi^2(2) = 2937.3$<br>$p < .001$	$\beta = 1.01$<br>$SE = 0.03$<br>$\chi^2(2) = 2445.7$<br>$p < .001$	$\beta = 0.39$<br>$SE = 0.05$<br>$\chi^2(1) = 69.7$<br>$p < .001$
**Heuristic**	**Explicit Probe**	**Implicit Probe**	**Teleportation Question**
QCut [3]	$\beta = -0.15$<br>$SE = 0.01$<br>$\chi^2(2) = 1177.3$<br>$p < .001$	$\beta = -0.21$<br>$SE = 0.01$<br>$\chi^2(2) = 1025.3$<br>$p < .001$	$\beta = 0.07$<br>$SE = 0.04$<br>$\chi^2(1) = 2.8$<br>$p = .095$
Degree Cent. (log)	$\beta = 0.71$<br>$SE = 0.02$<br>$\chi^2(2) = 1811.1$<br>$p < .001$	$\beta = 0.64$<br>$SE = 0.02$<br>$\chi^2(2) = 1439.2$<br>$p < .001$	$\beta = 0.46$<br>$SE = 0.05$<br>$\chi^2(1) = 91.9$<br>$p < .001$
Betweenness Cent. (log)	$\beta = 0.89$<br>$SE = 0.02$<br>$\chi^2(2) = 4321.3$<br>$p < .001$	$\beta = 0.85$<br>$SE = 0.02$<br>$\chi^2(2) = 3629.0$<br>$p < .001$	$\beta = 0.58$<br>$SE = 0.04$<br>$\chi^2(1) = 229.1$<br>$p < .001$
State Occupancy (log)	$\beta = 1.02$<br>$SE = 0.03$<br>$\chi^2(2) = 2444.8$<br>$p < .001$	$\beta = 0.92$<br>$SE = 0.03$<br>$\chi^2(2) = 2096.2$<br>$p < .001$	$\beta = 0.38$<br>$SE = 0.05$<br>$\chi^2(1) = 74.4$<br>$p < .001$

Table A2. Estimated coefficients with standard errors from hierarchical multinomial regression predicting probe trial choice, where models include regressors for the icon shown. Likelihood ratio test is performed for the addition of fixed and random effects for subgoal predictions onto a null model containing only regressors for presented icons.

Algorithm	β	SE	Likelihood ratio test
RRTD-IDDFS	1.80	0.02	$\chi^2(2) = 3901.1$, $p < .001$
RRTD-BFS	4.38	0.07	$\chi^2(2) = 3335.0$, $p < .001$
RRTD-RW	0.35	0.02	$\chi^2(2) = 471.5$, $p < .001$
Solway et al. (2014) [1]	0.68	0.01	$\chi^2(2) = 2786.9$, $p < .001$
Tomov et al. (2020) [2]	0.96	0.02	$\chi^2(2) = 2545.5$, $p < .001$
QCut [3]	-0.26	0.01	$\chi^2(2) = 838.7$, $p < .001$
Degree Cent. (log)	0.68	0.01	$\chi^2(2) = 1585.7$, $p < .001$
Betweenness Cent. (log)	0.99	0.01	$\chi^2(2) = 4543.3$, $p < .001$

The influence of graph complexity and structure on subgoal choice.

We consider the influence of two graph measures on reports of subgoal choice, in order to understand how graph complexity and structure influence the ability of participants to identify subgoals or plan hierarchically. We compare graph measures to the subgoal choice count, or the number of times participants reported the use of a subgoal instead of the goal in the Explicit Probes. The subgoal choice count is also compared with navigation behavior in the main text.

First, we consider how the complexity of a graph might influence subgoal use. We note that participants may be influenced in either direction: A complex graph could be more difficult to learn, impeding identification of hierarchy. On the other hand, a complex graph may incentivize the use of hierarchy to support efficient planning. We use the number of edges per graph as a simple proxy for complexity. When a graph is represented as an *adjacency list*—a list of all the edges in the graph—it has a description length that is directly related to the number of edges. We find that the edge count and the average per-graph subgoal choice counts are uncorrelated ($r = 0.04$, $p = .821$). This analysis suggests subgoal choice identification and use is minimally impacted by graph complexity as measured by edge count.

The second measure we consider is related to structural properties of the graph—the spectral gap. We describe the spectral gap at length below, but briefly note that the spectral gap corresponds to a measure of graph modularity—graphs with a small spectral gap should be more modular and those with a large spectral gap should be more densely connected. Beyond this relationship to graph modularity, it has a relationship to the *mixing time*, or the rate at which a random walk converges to a stationary distribution [4]. We find a small, but non-significant, correlation between the spectral gap and the average per-graph subgoal choice count ($r = -0.22$, $p = .246$). A negative correlation means that participants selected subgoals more often for modular graphs (smaller spectral gap), and less often for densely connected graphs (larger spectral gap).

Testing whether icons influence participant choice analysis

We test whether icons influence participant choice by extending the hierarchical multinomial analyses of probe choice in the main text. We first evaluate whether subgoal predictions meaningfully explain the same choice data pooled across probe types when added to a null model containing fixed effects and per-participant random effects for the dummy-coded icon regressors. We use a likelihood ratio test for the addition of fixed and random effects for subgoal predictions to this null model—this test mirrors the likelihood ratio tests in the main text, besides the change in the null model. Across all models, we find that the addition of subgoal predictions is justified (Table A2). Now, we evaluate whether the overall level of predictivity among models is impacted by the presence of the icon regressor in Fig A3. We find that the qualitative results observed in the main text are conserved. This indicates that icons have minimal influence on the results presented in the main text.

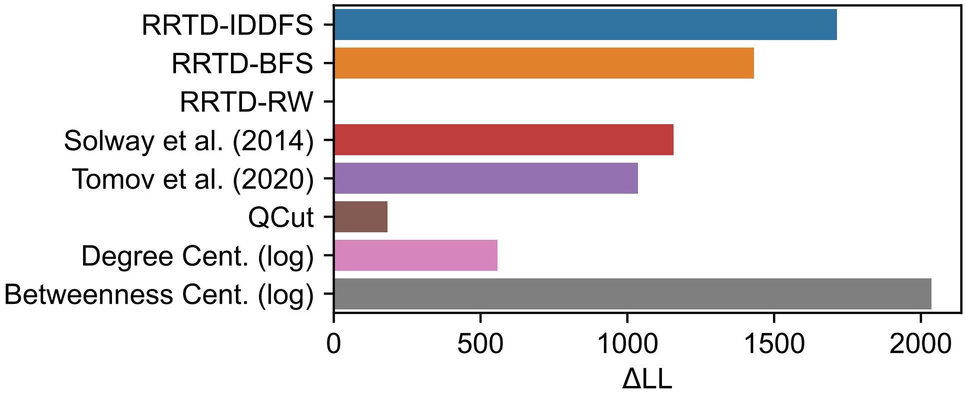

Fig A3. Predicting probe trial choice using hierarchical multinomial regression using subgoal predictions and additional regressors for the icon displayed. Despite the inclusion of the icon regressor, these qualitative results mirror those in the main text. Log likelihood (LL) is relative to the minimum model LL. Larger values indicate better predictivity.

Predicting navigation response times

We first examined how participant response times vary during navigation, focusing on the difference between response times at self-reported subgoals and other states. For simplicity, we examine navigation trials with optimal behavior and an associated Explicit Probe trial where participants reported a subgoal that they also visited during navigation. First, we found that participants respond most slowly at the initial state ($M = 5.36s$, $SD = 7.58$) when compared to their responses at their self-reported subgoals ($M = 2.00s$, $SD = 1.38$; $t(789.8) = 11.9$, $p < .001$) or other states ($M = 1.70s$, $SD = 1.12$; $t(773.4) = 13.0$, $p < .001$). We also found that participants respond more slowly at their self-reported subgoal than at other states ($t(1423.9) = 4.6$, $p < .001$). We found similar results for the Implicit Probe trials. These findings could indicate that participants defer making detailed plans until they are necessary—that is, after reaching a subgoal, participants figure out how to reach the subsequent subgoal or goal.

Based on this result, we used model predictions for subgoals to predict response times during navigation. We analyzed every state choice during long navigation trials where participants took an optimal path. In order to focus on response times at subgoals compared to other states, we excluded the initial state to avoid any effect from initial planning. We found results that were largely qualitatively consistent with those observed elsewhere in the study (Fig A4 and Table A3). The models that fit the observed data best included Betweenness Centrality and RRTD-IDDFS. Most models had positive coefficients, consistent with the idea that response times should be higher at states that are more likely to be subgoals. However, two other models also fit the data well: Degree Centrality and QCut. The efficacy of Degree Centrality as a predictor suggests that people simply take longer to act at states that have greater degree (i.e. states that have more edges). The efficacy of QCut is more difficult to interpret in the context of our other results, particularly since it has a negative coefficient and is the best fit to the data.

Hierarchical linear regression analyses were fit using `lme4` and predicted the logarithm of participant response times in seconds. The regression models included an intercept and slope for subgoal predictions as effects at three levels: fixed effects, random effects for each graph, and random effects for each participant. In addition, they included a fixed effect of the trial number, in order to fit differences resulting from learning over the course of trials. Because the regression models have identical effect structure, we compare them by log likelihood (LL).

A formal relationship between QCut and RRTD-RW

We prove a formal relationship between QCut [3, 5] and RRTD-RW for tasks based on undirected graphs. We show that a low-rank spectral approximation of RRTD-RW is equivalent to QCut for regular graphs. We use the notation introduced in the main text, recapitulated briefly here. An undirected graph has nodes $\mathcal{V}$ and edges $\{i, j\} \in \mathcal{E}$ between nodes i and j. We define $n = |\mathcal{V}|$ and $m = |\mathcal{E}|$. The adjacency matrix $A_{ij} = 1$ if

Table A3. Estimated coefficients with standard errors from hierarchical linear regression predicting response times for action choices during navigation trials.

Algorithm	β	SE	Likelihood ratio test
RRTD-IDDFS	0.07	0.03	$\chi^2(8) = 6074.8$, $p < .001$
RRTD-BFS	0.34	0.12	$\chi^2(8) = 5932.4$, $p < .001$
RRTD-RW	0.03	0.02	$\chi^2(8) = 6012.7$, $p < .001$
Solway et al. (2014) [1]	0.03	0.01	$\chi^2(8) = 5964.5$, $p < .001$
Tomov et al. (2020) [2]	0.03	0.01	$\chi^2(8) = 5966.5$, $p < .001$
QCut [3]	-0.05	0.01	$\chi^2(8) = 6182.9$, $p < .001$
Degree Cent. (log)	0.04	0.01	$\chi^2(8) = 6053.6$, $p < .001$
Betweenness Cent. (log)	0.05	0.01	$\chi^2(8) = 6028.5$, $p < .001$

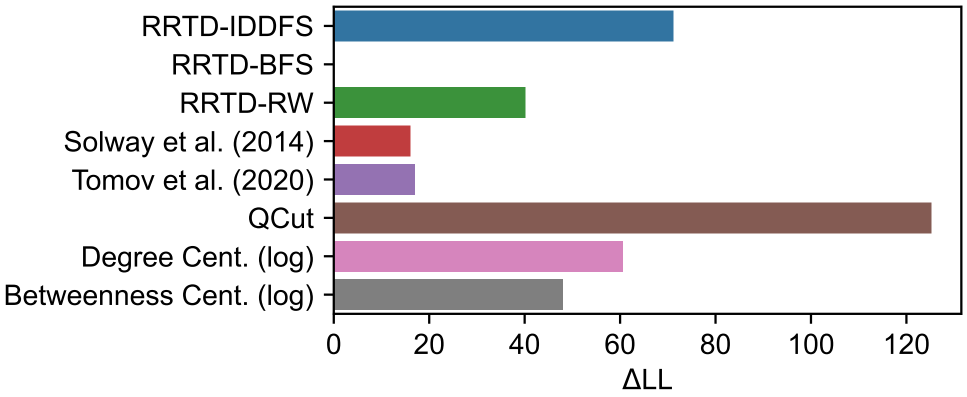

Fig A4. Predicting navigation response times using hierarchical linear regression. Log likelihood (LL) is relative to the minimum model LL. Larger values indicate better predictivity.

$\{i, j\} \in \mathcal{E}$ and 0 otherwise. The degree of a node i is $d_i = \sum_j A_{ij} = \sum_i A_{ij}$ and the degree matrix $D = diag(d)$. For simplicity, we assume a uniform distribution over tasks $p(s_0, g) = \frac{1}{|\mathcal{S}|^2}$ and optimize for single subgoals z, so that $\mathcal{Z} = \{z\}$.

To start, we introduce notation from [4] for the spectral definition of the hitting time. The matrix $N = D^{-\frac{1}{2}} A D^{-\frac{1}{2}}$ is symmetric, so it can be decomposed into eigenvalues λ_k and eigenvectors v_k in the definition $N = \sum_{k=1}^{n} \lambda_k v_k v_k^T$, with $1 = \lambda_1 > \lambda_2 \geq \cdots \geq \lambda_n \geq -1$. We denote the ith element of an eigenvector v_k as v_{ki}. The hitting time $H(s, z)$ can be computed using this spectral decomposition of N [4]—the hitting time from a state s to z and back from z to s is

$$H(s, z) + H(z, s) = 2m \sum_{k=2} \frac{1}{1 - \lambda_k} \left(\frac{v_{kz}}{\sqrt{d_z}} - \frac{v_{ks}}{\sqrt{d_s}} \right)^2$$

Since we assume a uniform distribution over tasks, we can simplify the value of task decomposition $V(\mathcal{Z})$ using the special case of $V_{\mathcal{Z}}^{g}(s_0)$ for RRTD-RW in Eq 4 in the main text.

$$
\begin{aligned}
V(\mathcal{Z}) &= \sum_{s_0,g} p(s_0,g) V^g_{\mathcal{Z}}(s_0) \\
&= -\frac{1}{n^2} \sum_{s_0,g} H(s_0,z) + H(z,g) \\
&= -\frac{1}{n} \left(\sum_{s_0} H(s_0,z) + \sum_{g} H(z,g) \right) \\
&= -\frac{1}{n} \sum_{s} H(s,z) + H(z,s)
\end{aligned}
$$

Having simplified, we can straightforwardly incorporate the spectral form of the hitting time.

$$
V(\mathcal{Z}) = -\frac{2m}{n} \sum_{k=2}^{n} \frac{1}{1-\lambda_k} \sum_s \left(\frac{v_{kz}}{\sqrt{d_z}} - \frac{v_{ks}}{\sqrt{d_s}} \right)^2 \tag{A1}
$$

We now apply this definition to develop a formal justification for QCut.

Justifying the choice of eigenvector in QCut

To approximate $V(\mathcal{Z})$ using a single eigenvector, a reasonable choice is v_2 since it has largest weight $\frac{1}{1-\lambda_k}$. We can compare this choice of eigenvector to that of QCut, since the matrices N and $\mathcal{L}_{\text{sym}}$ share eigenvectors because $\mathcal{L}_{\text{sym}} = I - N$. The eigenvalues e_k of $\mathcal{L}_{\text{sym}}$ are $e_k = 1 - \lambda_k$ with $0 = e_1 < e_2 \leq \cdots \leq e_n \leq 2$, since $\mathcal{L}_{\text{sym}} v_k = (I - N) v_k = (1 - \lambda_k) v_k$. The QCut algorithm makes use of the eigenvector of $\mathcal{L}_{\text{sym}}$ with the second smallest eigenvalue [3, 6]—the second smallest eigenvalue is e_2, which means the eigenvector used by QCut is v_2. Our approximation of $V(\mathcal{Z})$ based on the weight $\frac{1}{1-\lambda_k}$ thus provides an informal rationale for the choice of eigenvector v_2 in QCut. To provide a rationale for the threshold used in QCut, we further simplify this equation for regular graphs.

Justifying QCut thresholding in regular graphs

We can further connect RRTD-RW and QCut by limiting our consideration to regular graphs. We let d be the degree of nodes in a regular graph. For regular graphs, note that $\mathcal{L} = I - D^{-1}A = \mathcal{L}_{\text{sym}}$.

We first establish a helpful lemma: for each eigenvector v_k of $\mathcal{L}$, $\sum_s v_{ks} = 0$. Note that in general for matrices M where the columns j sum to zero (formally, $\sum_i M_{ij} = 0$), it is the case that $\sum_i (Mv)_i = \sum_{i,j} M_{ij} v_j = \sum_j v_j \sum_i M_{ij} = 0$. The columns j of $\mathcal{L}$ sum to zero, since $\sum_i \mathcal{L}_{ij} = \sum_i \left[I - D^{-1}A \right]_{ij} = 1 - \frac{1}{d} \sum_i A_{ij} = 0$. So, we know that $\sum_s (\mathcal{L}v)_s = 0$. In particular, $\sum_s (\mathcal{L}v_k)_s = \sum_s e_k v_{ks} = 0$, so $\sum_s v_{ks} = 0$.

We can now further simplify the above expression for $V(\mathcal{Z})$ since the v_k are unit vectors and $\sum_s v_{ks} = 0$ by the above lemma.

$$
\begin{aligned}
V(\mathcal{Z}) &= -\frac{2m}{dn} \sum_{k=2}^{n} \frac{1}{1-\lambda_k} \sum_s \left(v_{kz} - v_{ks} \right)^2 \\
V(\mathcal{Z}) &= -\frac{2m}{dn} \sum_{k=2}^{n} \frac{1}{1-\lambda_k} \sum_s v_{kz}^2 + v_{ks}^2 - 2 v_{kz} v_{ks} \\
V(\mathcal{Z}) &= -\frac{2m}{d} \sum_{k=2}^{n} \frac{1}{1-\lambda_k} \left(v_{kz}^2 + \frac{1}{n} \right)
\end{aligned}
$$

Based on the rationale in the previous section, we can approximate this expression using a single eigenvector v_2 as $\tilde{V}(\mathcal{Z}) \propto -v_{2z}^2$. This expression is maximized by subgoals with corresponding eigenvector entry closest to 0. In QCut, states are partitioned based on a threshold of their entry in an eigenvector—typically, values of 0 or the median are used.

Altogether, these results establish a connection between RRTD-RW and QCut. We assume a uniform task distribution, rank-one approximation based on the weight $\frac{1}{1-\lambda_k}$, and that a graph is regular. With these assumptions, we can give a rationale for QCut that uses v_2 with a threshold of 0. Connections between graph spectra, spectral clustering, and random walks have been noted in passing in previous research [6–8], though the authors are not aware of a result directly relating the models considered in this section.

Explaining the relationship between RRTD-RW and Degree Centrality

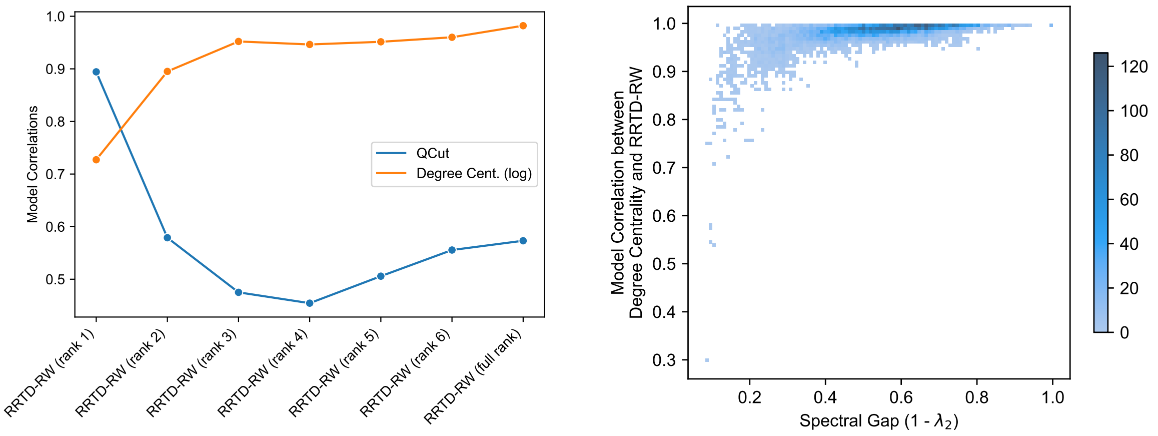

(a) **(b)**

Fig A5. a) Correlation of two models (QCut in blue and Degree Centrality in orange) plotted as a function of rank of a spectral approximation to RRTD-RW using the below formula for rank-k' spectral approximation. Correlations were computed as in Fig 4 in the main text. b) Bivariate histogram of the 11,117 eight-node graphs based on the spectral gap (1 - λ_2) and correlation between Degree Centrality and RRTD-RW. A small spectral gap is associated with the existence of a bottleneck, explaining the relative decrease in Degree Centrality/RRTD-RW correlation for those values.

The plot in Fig 4 in the main text validates our formal analysis by demonstrating an empirical result of modest correlation between QCut and RRTD-RW. However, Degree Centrality and RRTD-RW exhibit an even higher correlation which we examine in this section. A critical step in our above proof relating QCut and RRTD-RW requires a rank-one, spectral approximation to RRTD-RW. We examine how this approximation influences the relationships between models as the rank of the approximation is varied in Fig A5a, using this variant of Eq A1 to compute a rank-k' spectral approximation of RRTD-RW:

$$V(\mathcal{Z} \mid k') = -\frac{2m}{n} \sum_{k=2}^{k'} \frac{1}{1-\lambda_k} \sum_s \left(\frac{v_{kz}}{\sqrt{d_z}} - \frac{v_{ks}}{\sqrt{d_s}} \right)^2 .$$

In order to ensure eigenvectors with identical eigenvalues contribute equally, so as to avoid any influence relating to eigenvector order, we additionally group eigenvectors by eigenvalue and average their contributions with appropriate weight based on k'. We make sure to group eigenvalues that are only distinct due to small numerical errors.

We find that the relationship between QCut and RRTD-RW is highest when RRTD-RW is approximated with a single eigenvector, which is consistent with the rank-one approximation necessary in the proof. The correlation between QCut and RRTD-RW is also only higher than the correlation between Degree Centrality and RRTD-RW for the rank-one approximation. For ranks greater than one, Degree Centrality has the highest correlation to RRTD-RW, with the maximum correlation to the full-rank RRTD-RW. Our formal

analysis makes a stronger prediction for the case of regular graphs—in particular, QCut should be completely correlated with a rank-one approximation of RRTD-RW. When restricting attention to regular graphs, we find that QCut and rank-one RRTD-RW have a correlation of one, aligning with the formal analysis.

To conclude, we briefly and informally interpret the correlation between Degree Centrality and RRTD-RW by using the spectral gap $\lambda_1 - \lambda_2 = 1 - \lambda_2$ as a characterization of graph modularity. The spectral gap can be bounded by the Cheeger constant [4], a graph theoretic property related to the modularity of a graph—because of this connection, we will informally interpret the spectral gap as we would the Cheeger constant. In graphs with a small spectral gap, we expect more modularity with sparse connectivity between groups. In graphs with a large spectral gap, we expect the absence of obvious groups because of dense connectivity. In Fig A5b we plot the correlation between Degree Centrality and RRTD-RW against the spectral gap as a bivariate histogram. In graphs with large spectral gaps, we find that Degree Centrality and RRTD-RW are very correlated—This is consistent with intuitive predictions for a densely connected graph, where the most useful subgoals are those with higher degree because this means they are well-connected. In graphs with small spectral gaps, we find that Degree Centrality and RRTD-RW tend to be less correlated with greater variability. This is consistent with intuitive predictions for a modular graph, where a well-connected subgoal is less useful than a bottleneck.

References

1. Solway A, Diuk C, Córdova N, Yee D, Barto AG, Niv Y, et al. Optimal Behavioral Hierarchy. PLOS Computational Biology. 2014;10(8):1–10.

2. Tomov MS, Yagati S, Kumar A, Yang W, Gershman SJ. Discovery of hierarchical representations for efficient planning. PLOS Computational Biology. 2020;16(4):1–42.

3. Şimşek Ö, Wolfe AP, Barto AG. Identifying useful subgoals in reinforcement learning by local graph partitioning. In: Proceedings of the 22nd International Conference on Machine Learning; 2005.

4. Lovász L. Random walks on graphs: a survey. In: Miklós D, Sós VT, Szönyi T, editors. Combinatorics, Paul Erdös is Eighty. vol. 2. Budapest: János Bolyai Math Society; 1993. p. 353–397.

5. Menache I, Mannor S, Shimkin N. Q-Cut—Dynamic discovery of sub-goals in reinforcement learning. In: European Conference on Machine Learning. Springer; 2002. p. 295–306.

6. Shi J, Malik J. Normalized cuts and image segmentation. IEEE Transactions on pattern analysis and machine intelligence. 2000;22(8):888–905.

7. Stachenfeld KL, Botvinick MM, Gershman SJ. The hippocampus as a predictive map. Nature Neuroscience. 2017;20(11):1643–1653.

8. Von Luxburg U. A tutorial on spectral clustering. Statistics and computing. 2007;17(4):395–416.
