# Supplementary material for: Humans decompose tasks by trading off utility and computational cost
Source: PLoS Comput Biol. 2023 Jun 1;19(6):e1011087. doi: 10.1371/journal.pcbi.1011087 (PMC10234566; doi:10.1371/journal.pcbi.1011087)
Supplement: Attachment — Submitted filename: response-to-reviewers.pdf [file pcbi.1011087.s002.pdf]

Reviewer 1

We are very grateful to the reviewer for carefully reading of our manuscript and providing helpful comments and perspectives. Based on this feedback, we have added additional discussion and analyses, which we hope will serve to address the reviewer's concerns.

Below, the reviewer's original comments are in italics and our responses proceed in non-italics. Quotes from the updated manuscript are in blue.

The manuscript proposes a normative computational account of subgoal formation, which aims to integrate how the subgoals are chosen with the ways they are subsequently used, in the class of problems that translates into pathfinding problems on graphs. The authors implement a human behavioural experiment to assess the predictive power of the computational account and conclude that one of the graph search algorithms, one that closely corresponds to an interesting heuristic, corresponds to human choices of subgoals the best. In my opinion the paper is very thorough, thoughtful and very successful in integrating computational insight with empirical observation in a rather subtle domain. I think it would be a great addition to the scientific record - however, I believe addressing the following concerns would make it easier for readers to correctly contextualise the presented results:

There are three problem classes defined by the paper: the one described by the mathematical formalism of the model, the one implemented in the experiment, and the one from which the examples in the text are given. I think the text basically treats all these three to be the exact same, and I think they are not. I don't necessarily think that this is a problem - abstracting away some aspects of the experimental setup and addressing a more general set of phenomena in the examples might even be desired - but I think writing very clearly about these differences is important to help the reader see what the results really mean. My first two questions address these differences.

1. The mathematical formalism assumes T to be known, and to be accessed locally (i.e. no searching from the goal backwards). This

is fortunate since learning T would almost certainly be entangled with the choice of Z making the formalism way too complicated. However, in the experiment, people have to learn T from scratch, and aren't expected to have a noiseless version of it at any point. In the navigation trials, this is compensated by the local information about edges that can be said to correspond to the local access in the model - but there people are making actual moves on the graph, not merely mental ones, as e.g. in the given example of chess. And in the probes, where they presumably are making mental simulations, they presumably not only learned T already (albeit noisily) but also already constructed Z - thus people aren't really 'planning' while constructing the subgoals, but rather 'exploring'. All this might be perfectly fine, it just gave me pause not to see these points addressed more directly.

We thank the reviewer for this keen perspective. We agree that these differences are critical to note, since the formal alignment between the examples, experiment, and models are imperfect. We have called attention to this issue in the *Discussion*, noting this difference between how participants have learned task structure during the probe trials and navigation trials:

> An additional difference during navigation trials is that participants are still learning the task structure and shown connections from the current state. By contrast, during the probe trials, participants are unable to see any connections and must rely solely on what they have learned. These subtle differences may reduce the relationship between these two trial types. They are also different from the formal framework which assumes perfect knowledge about task structure—because this could influence task decomposition, we note a relevant extension below. The effect of this difference could be studied by introducing a separate experimental phase where participants are trained on the task structure directly, outside the context of navigation—the first experiment in Solway et al. (2014) contains a similar phase, but had limited navigation trials.

As mentioned above, we note a relevant extension to the framework later in the *Discussion*:

A particularly interesting direction could incorporate model learning (as in the Dyna architecture [Sutton and Barto, 2018]) across tasks in order to explain the influence of task learning on task decomposition.

The reviewer's concern about when $\mathcal{Z}$ and plans are constructed is related to a general issue regarding efficient computation of resource-rational objectives. These objectives require an estimate of computational cost, but estimating that cost in a naive fashion is usually more computationally costly than the original objective. We discuss this issue further in the second reviewer's fifth major question (Page 28).

> *2. The text of the manuscript gives various real-world examples of problems, most notably that of physical navigation, choosing landmarks (the café) as subgoals. I think the café example works through a different mechanism: I'd choose it as a subgoal because I already know how to get there, i.e. there is an existing (habitualised) policy I can reuse, and not because it will make planning cheaper now. Habits seem to be a consideration orthogonal to this paper, but maybe I'm failing to see a connection. Furthermore, in tasks like physical navigation people seem to heavily rely on distance-from-goal or directional heuristics, both available due to the existence of an embedding into a feature space. This could result in something like an IDA* search algorithm instead of the ones discussed here. No such embedding is assumed either in the model or the experiment - in fact it's explicitly avoided. The example of navigating in the dark is closer to the experiment, however not the model. Board game examples are closer to the model, as they involve planning in symbolic spaces (but one has to ignore heuristic-generating feature embeddings for e.g. chess). Cooking might be the example I find the most fitting for the model.*

We thank the reviewer for carefully considering the alignment between our examples and modeling framework. We have taken a few actions in order to address these issues.

First, in the closing paragraph of *A Formal Framework for Task Decomposition*, we take care to note this divergence in search algorithms between

the real-world examples and the brute-force approach our manuscript focuses on:

> The formal presentation of our framework considers subgoal choice with intentionally restricted algorithms: brute-force search methods that exclude problem-specific heuristics to accelerate planning. However, the examples in this section (navigating to the post office via the café, navigating in a place with low visibility) likely rely on algorithms that incorporate heuristics, particularly related to spatial navigation. While outside the scope of this manuscript, our framework can flexibly incorporate any search algorithm that can define an algorithmic cost, including those that make use of heuristics. For example, in a previous theoretical study we applied an early version of our framework to task decomposition in the Tower of Hanoi by using A* Search [Ghallab et al., 2016] with an edit distance heuristic [Correa et al., 2020].

We still think the examples based on spatial navigation are valuable because they are very concrete and easy for readers to visualize, as in our motivating gridworld-like figure.

We strongly agree with the reviewer's concern about policy reuse. Habits are an entirely orthogonal concern, and expressly avoided in our formulation. We have made a brief note about policy reuse when discussing extensions of the model in the *Discussion*:

> At present, our framework assumes planning for tasks occur independently, avoiding the reuse of previous solutions to subtasks. Despite this absence, our framework still predicts a normative benefit for problem decomposition. However, research has found that people learn hierarchically, exhibiting neural signatures consistent with those predicted by hierarchical reinforcement learning theory [Ribas-Fernandes et al., 2011]. Further research could relax the independence between task solutions by explicitly reusing solutions (as in [Solway et al., 2014]) or turning to formulations based on reinforcement learning [Harb et al., 2018], particularly those designed for a distribution of goal-directed tasks with shared structure as studied in this manuscript [Nasiriany et al., 2019]. A

> particularly interesting direction could incorporate model learning (as in the Dyna architecture [Sutton and Barto, 2018]) across tasks in order to explain the influence of task learning on task decomposition.

For the purpose of this example, we think habits and policy reuse should not be emphasized. To do so, we have made a small change in the language of the example, from

> Maybe the café you stop by every morning before work...

to a version that deemphasizes habit:

> Maybe the café you sometimes stop by before work...

> *3. The Discussion mentions in passing that this work is complementary to option discovery. This seems to be an important point to address, and although I myself am not well versed enough in the options literature to be able to tell the exact relationship, I'd be very interested how exactly is it complementary. In fact, I'd probably mention this in the introduction as well instead of only at the very end.*

We thank the reviewer for noting the importance of more clearly relating our framework and research to the options framework. In response to this comment, we have explicitly described how our framework relates to the options framework at the end of *A Formal Framework for Task Decomposition*:

> Our framework also considers a constrained set of task decompositions that consist of individual subgoals. In comparison, the influential *options framework* [Sutton et al., 1999] defines a more general set of hierarchical task decompositions, in which, for instance, subtasks can be defined by the subgoal of reaching any one out of a set of states and subtask completion can be nondeterministic. However, finding such subtasks remains a challenging problem in machine learning, so by focusing on the simpler problem of selecting a single subgoal we are able to make a

significant amount of progress in understanding a key component of how humans plan. While not yet fully explored, our formalism can be extended to encompass broader types of hierarchy (and also varied search algorithms). For instance, another theoretical study adapted our framework to support more varied kinds of hierarchical structure by incorporating abstract spatial subgoals in a block construction task [Binder et al., 2021].

---

4. Relatedly, the learning problem in this paper is formalised similarly to goal-directed reinforcement learning. In particular, this paper looks at defining subgoals in such a setting: https://arxiv.org/abs/2106.01404. It might worth relating to this literature as well.

We thank the reviewer for this reference to goal-directed reinforcement learning. In the *Discussion*, we have made explicit reference to this reinforcement learning setting (reference italicized):

> Further research could relax the independence between task solutions by explicitly reusing solutions (as in [Solway et al., 2014]) or turning to formulations based on reinforcement learning [Harb et al., 2018], *particularly those designed for a distribution of goal-directed tasks with shared structure as studied in this manuscript [Nasiriany et al., 2019].*

---

5. Why is IDDFS an intuitive choice despite the large computational cost depicted in Fig 1b? It has presumably been proposed previously because of some favorable property, was it good memory complexity? If so, would it also make sense to look at how subgoals reduce memory costs?

We thank the reviewer for bringing up this point about the relative cost of IDDFS in Fig 1b. To address this issue we have added additional discussion. IDDFS does have smaller memory complexity than BFS, since it only tracks visited states along the current trajectory, while BFS tracks all states that have previously been considered. In classical planning, IDDFS is often

preferred to BFS for this reason, since furthermore BFS and IDDFS have the same form of runtime scaling with the size of the graph (both $O(b^d)$, though of course with a larger constant multiplier for IDDFS). We draw explicit attention to this at the end of the *Action-level Planning* subsection:

> While only noted in passing above, each of these algorithms makes subtle trade-offs between run-times, memory usage, and optimality. Focusing on BFS and IDDFS, the two optimal algorithms, we briefly examine these trade-offs. BFS visits states at most once, but requires remembering every previously visited state; by contrast, IDDFS will revisit states many times (i.e. greater run-time compared to BFS), but only has to track the current candidate plan (i.e. smaller memory use compared to BFS). In effect, IDDFS increases its run-time to avoid the cost of greater memory use. We briefly return to this point below when examining algorithm run-times.

We also discuss this at the end of *A Formal Framework for Task Decomposition*:

> The gap in run-time between IDDFS and BFS might make IDDFS seem inefficient—however, as noted in the algorithm descriptions above, IDDFS makes a trade-off of increased run-time in order to decrease memory usage. While outside the scope of the current manuscript, our formulation can be extended to incorporate other resource costs like memory usage in order to study how they influence task decomposition.

Relatedly, we note in our response to the reviewer's second question (Page 3) that a key strength of our framework is the ability to incorporate any algorithm that can define a search cost.

Finally, since our framework can be extended by incorporating additional computational costs, an intriguing future direction could integrate memory costs into this modeling framework. To the best of our knowledge, this is a relatively understudied topic, but we hope that our results might justify further inquiry along these lines. We briefly mention this in the *Discussion*:

> Another direction could explore other resource costs like memory use, motivated by the relatively low memory use of IDDFS discussed above.

6. Do Fig 1c-e show the costs incurred during one particular run of each of these algorithms? Or why is e.g. the random walk not symmetric around the start state? These panels are also a bit far from the part of the text that describes them, moving them closer, maybe on a separate figure could streamline the reading experience.

We are grateful for the reviewer's attention to detail here. We were able to address this issue, which arose because our grid implementation—used only for this figure—had the complete set of movements in the four cardinal directions for all states. Since some directions have no effect at a border, this meant that a random walk might get "stuck" at a border because some actions kept it in place. We have modified the grid implementation to avoid this issue. The random walk is implemented analytically, so that was unrelated to this issue.

The figure has been updated in the text. The RW without hierarchy appears as follows:

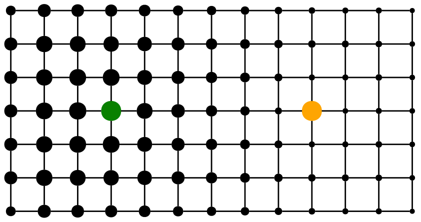

And RW with hierarchy is as follows:

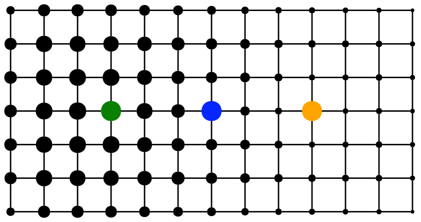

We have also split the figure so that the toy example is closer to the text where it is referenced. We have separately added additional context to the figure, in the hopes that it will be easier to understand without needing to reference the text.

Caption: State-specific search costs of the algorithms. The depicted task requires navigating on a grid from the start state

> (green) to the goal state (orange) with fewest steps. Each column corresponds to a different algorithm and demonstrates two scenarios—*Top*: Search cost without subgoals, *Bottom*: Search cost when using the path midpoint as a subgoal (blue). We define the search cost as the number of iterations required for the search algorithm to find a solution. Larger states were considered more often during the search algorithm, resulting in greater search cost.

> *7. In the experiment could stimulus salience distort the results in any way? E.g. is the red balloon overrepresented in the choice of subgoals?*

We thank the reviewer for noting this potential confound. We would first note that while icon effects are plausible, the assignment of icons to states was pseudorandomized across subjects (though not exactly counterbalanced), which mitigates many concerns about whether such effects would confound our broader conclusions. To investigate this formally, we added an analysis in the *Appendix* in *Testing whether icons influence participant choice analysis* to investigate whether the inclusion of icon as a regressor in our probe choice models has any impact on our observed results:

> We test whether icons influence participant choice by extending the hierarchical multinomial analyses of probe choice in the main text. We first evaluate whether subgoal predictions meaningfully explain the same choice data pooled across probe types when added to a null model containing fixed effects and per-participant random effects for the dummy-coded icon regressors. We use a likelihood ratio test for the addition of fixed and random effects for subgoal predictions to this null model—this test mirrors the likelihood ratio tests in the main text, besides the change in null model. Across all models, we find that addition of subgoal predictions is justified (Table in manuscript). Now, we evaluate whether the overall level of predictivity among models is impacted by the presence of the icon regressor in Figure (below). We find that the qualitative results observed in the main text are conserved.

This strongly suggests that icons have minimal influence on the results presented in the main text.

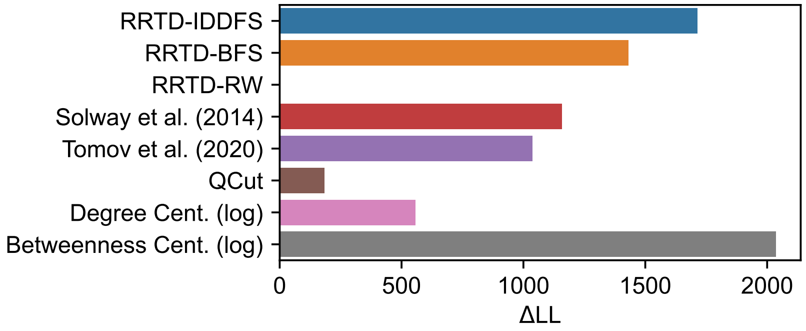

Caption: Predicting probe trial choice using hierarchical multinomial regression using subgoal predictions and additional regressors for the icon displayed. Despite the inclusion of the icon regressor, these qualitative results mirror those in the main text. Log likelihood (LL) is relative to the minimum model LL. Larger values indicate better predictivity.

---

8. Would it be possible to make more direct comparisons between the number of steps people make in the navigation trials and the number of steps taken by the algorithms? Would such a comparison be meaningful given that for humans this is a learning phase as well?

We appreciate the reviewer drawing attention to this issue. It is one that we, the authors, have thought about over the course of preparing this manuscript, and find it important to consider. The nature of the behavioral experiment makes it difficult to quantitatively analyze navigation trial behavior in order to identify the search algorithms that people use. The main issue (which arises in many attempts to study planning through choice behavior) is that planning steps are presumptively covert, and do not correspond in any simple way with steps of overt behavior given by the plan that is ultimately produced. Indeed, many different search algorithms (for

instance, all optimal ones) will arrive at the same plan and identical choices. We discuss this at more length in the *Discussion*:

> A third limitation is that the navigation trials provide limited insight into the algorithms people are using to plan. While participants only see local connections during navigation, analogous to the local visibility search algorithms have, there are a number of reasons why their navigation behavior would be difficult to relate to the choice of search algorithm. The main issue is that planning steps are generally covert, and do not correspond in any simple way with steps of overt behavior given by the plan that is ultimately produced. Such covert planning is better suited, in future work, to being studied through process-tracing experiments [Callaway et al., 2022, Ho et al., 2022], think-aloud protocols, or by investigating neural signatures related to planning and learning [Liu et al., 2021]. It is possible that some aspects of planning are externalized in the current experiment, via exploration on navigation trials, but this is at best incomplete. For instance, participant behavior improves over the course of navigation trials, suggesting they are performing mental search instead of search via navigation. Also, the experiment restricts single-step movement to neighboring states, whereas by contrast, algorithms like BFS might plan over states in an order where subsequent states are not neighbors. Identifying the search algorithm participants use is critical for future studies, since our framework predicts that task decomposition is driven by the search algorithm used.

> *9. Eq 3: is the Z that maximises this formula taken here? I assume it is but it isn't stated explicitly.*

We have made sure to clarify this in the updated manuscript:

> The optimal set of subgoals $\mathcal{Z}^*$ for planning maximize this value, so $\mathcal{Z}^* = \operatorname{argmax}_{\mathcal{Z}} V(\mathcal{Z})$.

> *10. It is mentioned that the subjects were asked if they drew a map. Did any of them answer yes to this question?*

We thank the reviewer for drawing attention to this potential confound. As detailed in the results, we have added an analysis predicting subgoal choice, restricted to the subset of participants that did not draw a map.

> Since the experiment was designed to so that only local connections were visible during navigation trials but conducted via an online platform, in the closing survey we asked participants if they used a reference to the task structure besides the interface ("Did you draw or take a picture of the map? If you did, how often did you look at it?"). Participant responses were as follows: 603 participants selected "Did not draw/take picture", 65 selected "Rarely looked", 90 selected "Sometimes looked", and 48 selected "Often looked". In order to ensure the above results were not impacted, we ran the same analysis in the subset of participants ($N = 603$) that selected "Did not draw/take picture" and found qualitatively similar results (in the Appendix).

The following figure was included to support these results.

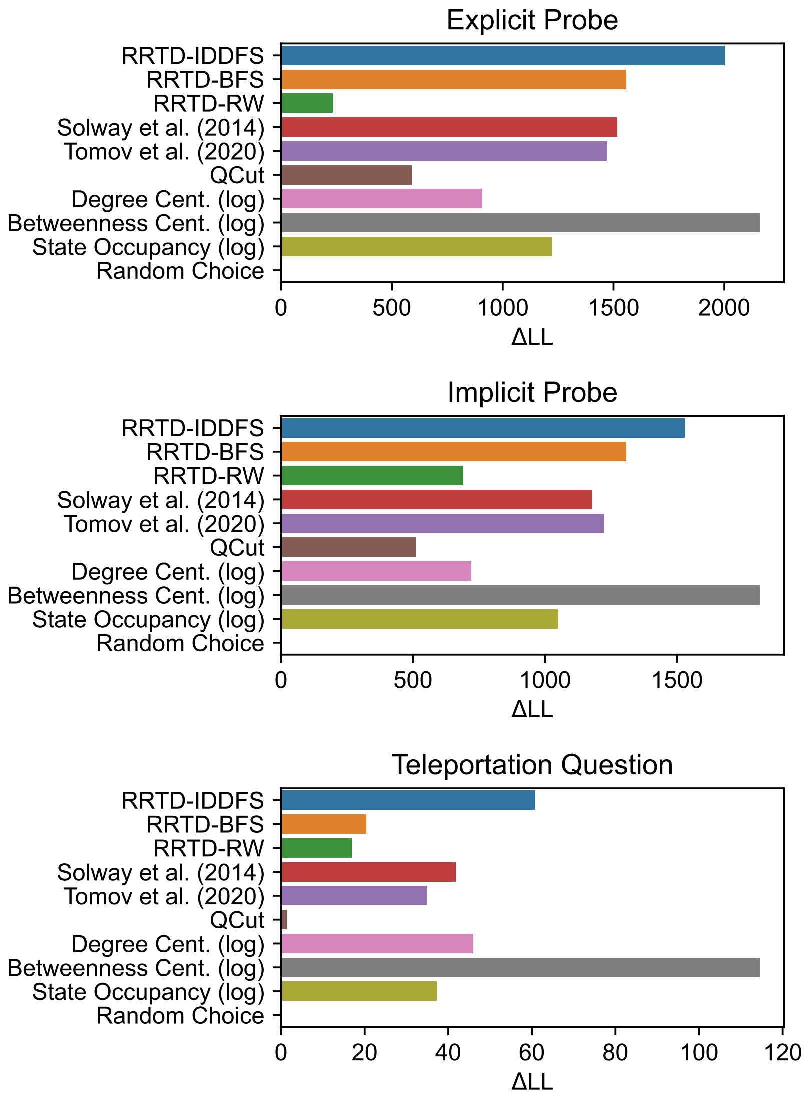

Caption: Comparison of probe choice behavior in the subset of participants ($N = 603$) that reported not using an additional visual aid during the experiment. Analysis is otherwise identical as that in main text, using mixed-effects multinomial regression to predict subgoal choice behavior for each subgoal probe. Log likelihood (LL) is relative to the minimum model LL for each probe. Larger values indicate better predictivity.

Reviewer 2

We are very grateful to the reviewer for carefully reading of our manuscript and providing helpful comments and perspectives. Based on this feedback, we have added additional discussion and analyses, which we hope will serve to address the reviewer's concerns.

Below, the reviewer's original comments are in italics and our responses proceed in non-italics. Quotes from the updated manuscript are in blue.

The paper describes behavioral predictions derived from a set of (normative) computational accounts and heuristics for the (resource-rational) decomposition of tasks in a graph-structured environment. For simulations, graph structures are selected such that differences between model predictions about which states should be sub-goals become qualitatively evident. Model predictions are qualitatively and quantitatively (using multinomial regression analyses) compared against human behavior from a large, pre-registered online study (N = 806). The authors report that human behavior in a graph-structured planning task involving explicit and implicit probe questions about sub-goals is most consistent with the use of a task decomposition heuristic (betweenness centrality) and – among the formal accounts – with a resource-rational model performing an iterative-deepening depth-first search on the graph structure.

The paper is well-written, addresses an interesting (and novel) research question and features a variety of well-crafted computational accounts of task decomposition that are motivated by a resource-rational perspective on planning. Predictions of previously considered formal accounts of planning from the literature are pitted up against these new algorithms – allowing for quantitative comparisons of the relative goodness of fit to observed human behavior. The novelty and strength of the present computational approach lies in the formalization of three nested levels of planning (action-level planning, subtask-level planning and task decomposition), and their optimization considering computational costs (limited resources).

The idea that humans (and potentially other cognitive systems) engage in resource-rational trade-offs during planning and decomposition of tasks is intriguing, and has far-reaching implications, even beyond the field of cognitive psychology/neuroscience.

While I enjoyed reading the paper and think it would be of much interest to the diverse readership of PLOS Computational Biology, I have a few comments that I would like to see addressed. These are mainly related to a potential confound in the behavioral task design (that should at least be discussed), the presentation/analysis of the behavioral data and the interpretation of the findings. I am very confident that the authors will be able to address my concerns.

Major questions and comments:

1.) I would like to see the human behavior unpacked and explored a bit more:

a. A figure for the observed associations between navigation trial performance and probe behavior could be used to illustrate these findings. This would help readers to get a better sense for the variability of performance across subjects and the data distributions at hand.

We thank the reviewer for this comment, and have incorporated an additional figure to address this concern. We have added a plot that shows the relationship between participant subgoal choice counts and navigation path length (Figure below). We hope this plot gives readers an enriched perspective into the data. In this revised version, the reported correlation is now negative, because we report "subgoal choice count" instead of "goal choice count" to clarify our exposition—these two features are perfectly negatively related, since participants either selected a subgoal or the goal for each of the Explicit Probe trials.

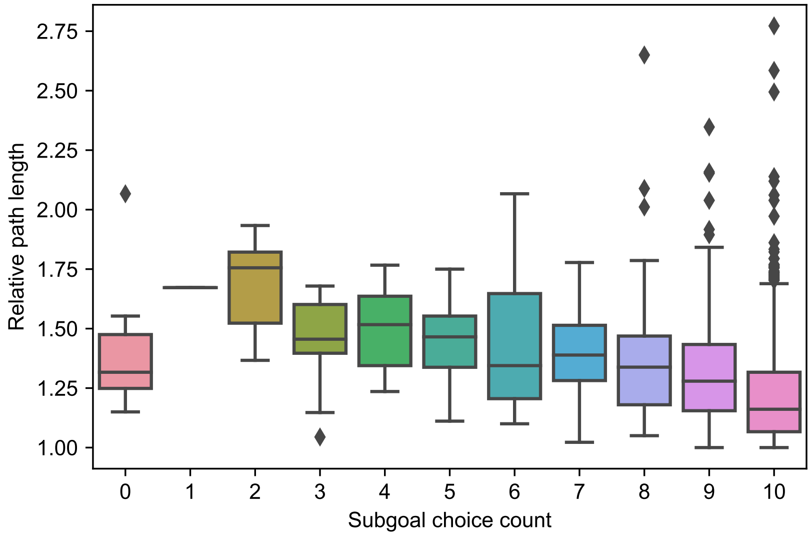

Caption: Participants that choose a subgoal instead of the goal more often in Explicit Probe trials have shorter average path length, relative to the optimal path length ($r = -0.29$, $p < .001$).

Unrelated to the above figure, while revisiting the code relating probe behavior to optimal path choice, we found that the reported results were based on an outdated analysis using an incorrect exclusion criteria. We have updated the results, which are qualitatively identical and quantitatively extremely similar.

---

b. In Figure 5a it is unclear how many participants performed choices on each of the depicted graphs. Please clarify this, and potentially consider adding a supplementary figure showing the results for the remaining 26 graph structures that were considered in the study.

We thank the reviewer for calling attention to this. We have updated the figure caption to incorporate the number of participants per graph

Each participant responded to a total of 21 subgoal probes and the number of participants per graph, from the top graph to the bottom graph, was 28, 26, 25, and 26.

and added the below figure to the supplement which displays the behavioral data for all 30 graphs.

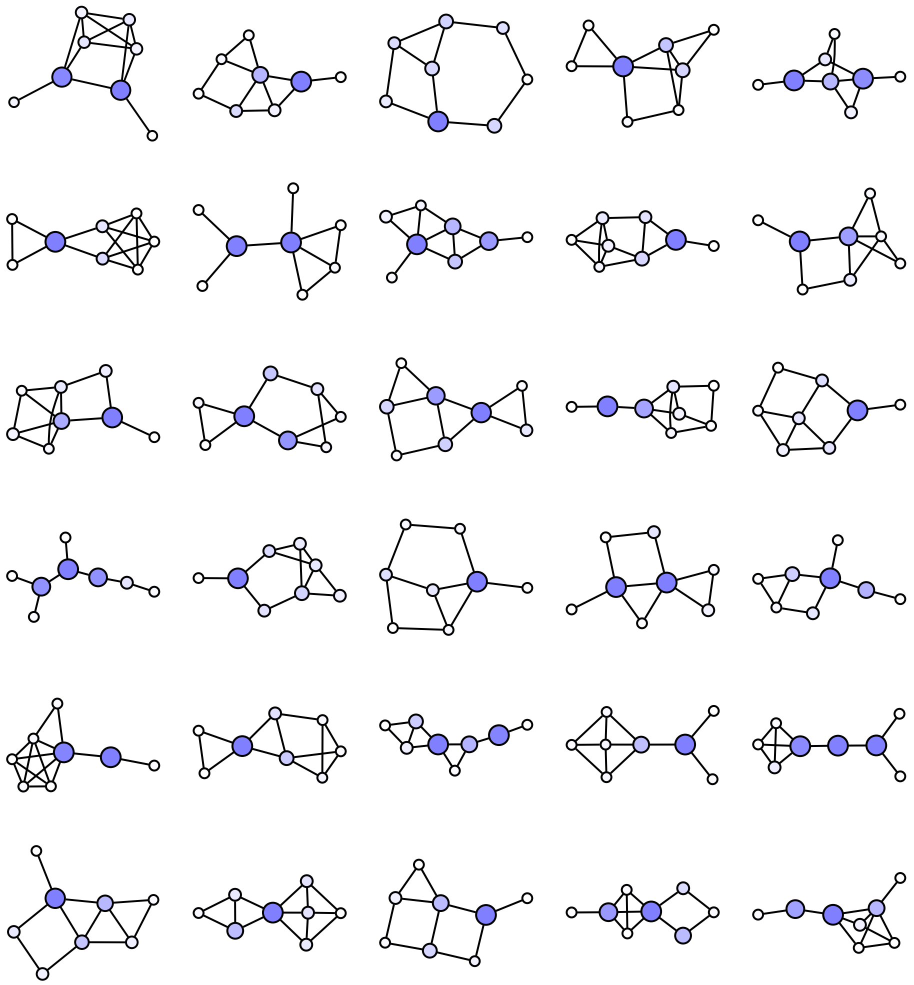

Caption: Visualization of participant behavior by graph. State color and size is proportional to subgoal choice, summed across participants and probe types. Participants per graph ranged from 21–30 and each participant responded to a total of 21 probe questions.

---

c. A potential confound that should be controlled for is the varying complexity of the employed graph structures. Is discovery and

usage of sub-goals further modulated by measures of graph complexity/minimum description length?

We appreciate this suggestion from the reviewer. In a new analysis in the appendix, we compare participant subgoal choice counts on the Explicit Probe to two different measures of graphs: one related to complexity and the other to structural properties. We are not aware of a clear consensus around measures of graph complexity, so we used these two quantities that we felt we could justify. We appreciated the opportunity to analyze the data from this perspective, and hope the reviewer finds the results interesting.

> We consider the influence of two graph measures on reports of subgoal choice, in order to understand how graph complexity and structure influence the ability of participants to identify subgoals or plan hierarchically. We compare graph measures to the subgoal choice count, or the number of times participants reported the use of a subgoal instead of the goal in the Explicit Probes. The subgoal choice count is also compared with navigation behavior in the main text.
>
> First, we consider how the complexity of a graph might influence subgoal use. We note that participants may be influenced in either direction: A complex graph could be more difficult to learn, impeding identification of hierarchy. On the other hand, a complex graph may incentivize the use of hierarchy to support efficient planning. We use the number of edges per graph as a simple proxy for complexity. When a graph is represented as an *adjacency list*—a list of all the edges in the graph—it has a description length that is directly related to the number of edges. We find that edge count and the average per-graph subgoal choice counts are uncorrelated ($r = 0.04$, $p = .821$). This analysis suggests subgoal choice identification and use is minimally impacted by graph complexity as measured by edge count.
>
> The second measure we consider is related to structural properties of the graph—the spectral gap. We describe the spectral gap at length below, but briefly note that the spectral gap corresponds to a measure of graph modularity—graphs with small spectral gap should be more modular and those with large spectral gap should be more densely connected. Beyond this relationship to

graph modularity, it has a relationship to the *mixing time*, or the rate at which a random walk converges to a stationary distribution [Lovász, 1993]. We find a small, but non-significant, correlation between the spectral gap and the average per-graph subgoal choice count ($r = -0.22$, $p = .246$). A negative correlation means that participants selected subgoals more often for modular graphs (smaller spectral gap), and less often for densely connected graphs (larger spectral gap).

---

d. Does behavioral performance improve across trials/repeated exposures to planning tasks?

We agree with the reviewer that it is an interesting question whether participant performance increased over the course of planning trials. As we mention in the response to the reviewer's next point (1e), we add one additional measure of performance (number of solutions that included a repeated state visit) that is consistent with an increase in behavioral performance. In our initial submission, we had also mentioned a few measures.

Even though the experimental interface obfuscated task structure by showing the task states in a random circular layout, participants became more effective from the first to the second half of training: "long" trials were solved more quickly (from 10.30s ($SD = 29.74$) to 7.60s ($SD = 11.19$)), with more efficient solutions (from 36% to 20% more actions than the optimal path; completely optimal solutions increased from 70% to 79%; solutions that included a repeated state decreased from 14% to 9%), and with decreased use of the map (on-screen duration decreased from 9.01s ($SD = 20.97$) to 2.98s ($SD = 12.66$); number of hovered states decreased from 5.43 ($SD = 8.70$) to 1.38 $SD = 3.99$; duration of state hovering decreased from 2.52s ($SD = 7.23$) to 0.60s ($SD = 9.04$)).

---

e. Is there evidence that participants learned the structure well-enough? What looks like absence of use of normative task decomposition could in fact be failure to acquire the structure. This

learning deficit could be assessed by investigating exploration behavior before setting sub-goals (entropy in cursor/mouse movement, return to previously visited states – over and above the reported control for state occupancy during navigation trials) as marker of how well the structure has been learned.

We agree with the reviewer's concern—a failure to learn task structure would confound our results. In order to address these concerns, we have added a few measures to our experimental results to explicitly note how participant behavior changes from the first to second half of navigation trials. These measures augment those in the initial submission (trials were solved more rapidly; solutions were shorter and a greater fraction were optimal; map was used less). The first additional measure is the fraction of solutions that contained a repeated state during navigation trials:

> solutions that included a repeated state decreased from 14% to 9%

We were also able to add two additional measures of map use, in addition to our initial measure of overall duration of map use. While neither measure directly addresses the reviewers suggestion for entropy of cursor movements, they give more insight into how participants used the between-trial map, and are related to cursor movements (since participants had to hover on states with the cursor in order to reveal state icons).

> number of hovered states decreased from 5.43 ($SD = 8.70$) to 1.38 $SD = 3.99$; duration of state hovering decreased from 2.52s ($SD = 7.23$) to 0.60s ($SD = 9.04$)).

Unfortunately, because we did not collect further cursor information, certain kinds of analysis (e.g. entropy of cursor coordinates) are not possible with this dataset.

Finally, we would direct the reviewer to the first reviewer's first point (Page 2), which is related. Our ideal-observer formalism assumes the graph structure T is known, and does not at present contemplate additional uncertainty about it, although this clearly arises in practice during learning in the experiment. We now highlight this difference between theory and experiment in discussion now, and note that future work could extend the formalism to incorporate such uncertainty.

> *f. Relatedly, was there evidence for (overall) longer reaction times on the task (e.g. longer planning duration, or time to complete a trial) for subjects choosing less optimal sub-goals (or no sub-goals at all), which would be expected if there are advantages of using a normative strategy to solve the task?*

We thank the reviewer for this analysis idea. We have added a simple analysis comparing participant subgoal choice counts to their response times during navigation trials:

> We also briefly examine the relationship between subgoal choice count on Explicit Probe trials and response times during navigation. We were unable to find evidence of a correlation between the subgoal choice count of a participant and their average log-transformed navigation trial duration ($r = 0.01$, $p = .858$). In order to understand how subgoal use influences response times, future studies should examine trial-level measures in appropriate experimental designs, an issue we remark on in the discussion.

As noted, we find no relationship between trial duration and subgoal use. This null result may be a result of the coarse participant-level measures we analyze, so more granular trial-level measures of response time and subgoal use may be necessary to determine whether subgoal use influences response times. We also think that future studies should probe these questions further with experimental designs that encourage a distinct phase of up-front planning—for instance, by prohibiting participant action during an explicit initial planning phase. We note these issues in the *Discussion*:

> Second, though we report analyses of participant response times, these analyses were not pre-registered and our experiment was not designed to assess response times. These findings should be reevaluated in experimental designs appropriate to assess response times. For example, we found that participants were slower to respond at their subgoals, suggesting they were planning at those states. However, our normative framework makes no prediction about *when* planning occurs. The framework only defines a resource-rational value for subgoals that can be used to simplify planning whether it happens before action or after reaching a subgoal. Future experiments could investigate this further

through manipulations to influence when planning is employed, like a timed phase for up-front planning or incentive for fast plan execution.

2.) Does task decomposition in the behavioral experiment occur "naturally", or is it induced by task instructions, e.g. "Plan how to get from A to B. Choose a location you would visit along the way," (lines 340-341), and subjects feel encouraged to do so; i.e. due to demand characteristics of the task? The authors should acknowledge and discuss this potential confound and its implications for the interpretation of the results. Additionally, it would be beneficial to point out that future studies should try to address this confound by using the same task but without explicitly prompting sub-goal use (and therefore task decomposition). This would allow to test whether the results are still aligned with the predictions of normative accounts and heuristics.

We understand the reviewer's concern and agree that this should be discussed in the manuscript. When designing the experiment, we were motivated by prior studies [Solway et al., 2014, Tomov et al., 2020, Balaguer et al., 2016] that reported participant behavior consistent with the formation and use of subgoals, even when unprompted. We draw particular notice to the first experiment in Solway et al. (2014), where participants report meaningful hierarchical structure when solely learning graph connections outside the context of planning. In our work, we thought it appropriate to prompt participants, since our focus is *which* subgoals participants choose, in contrast to *whether* participants plan hierarchically. However, we recognize that future work should investigate how experimental instructions influence behavior. We draw attention to this issue in the *Discussion*:

> The first [limitation] is that participants are encouraged to plan hierarchically ("It might be helpful to set subgoals" in Fig [...]). While this seems likely to have minimal impact on *which* subgoals participants choose, the main focus of this manuscript, it may impact *whether* participants plan hierarchically in the first place. Future studies intending to assess how people choose to plan hierarchically should consider avoiding prompts like this.

3.) It is unclear to me, how exactly the tested approximations/heuristics are more psychologically plausible and tractable for humans than the presented normative computations. As also discussed by the authors, betweenness centrality is computationally very demanding, given that all shortest paths of a given graph need to be computed and stored in memory to calculate the importance of each node using this heuristic. Relatedly, I would like to encourage the authors to include a section elaborating on how these heuristics (and potentially the computations used in the normative accounts) might plausibly be implemented by humans/brains (i.e. how cognitively and also how biologically plausible their implementation is). The authors acknowledge that even simpler heuristics than the ones considered here could be used by participants. I think it would be beneficial to elaborate more on what these simpler, more tractable heuristics may be. For example, simple count-based strategies, keeping track of the number of edges of and how often each node occurred in each query about start and end node seem to be a more tractable approximation (akin to something like a successor representation).

We thank the reviewer for voicing this concern. We agree that Betweenness Centrality is not trivial to estimate, and think it is interesting to discuss and investigate how it might be approximated by people. We have taken a few steps to address this issue, by discussing the issue of tractable estimation earlier in the text and clearly relating a trivial heuristic we examine (state occupancy) to another heuristic (betweenness centrality). While we were able to rule out the use of this trivial heuristic, we note that future research should give more attention to the question of how people might be approximating quantities such as Betweenness Centrality.

We have added additional discussion in the closing paragraph of *Comparing Accounts of Task Decomposition* that draws attention to the issue of tractable estimation of Betweenness Centrality, drawing notice to a simple memory-based strategy tracking visit rate, or state occupancy:

> The second approach might approximately optimize the objective by using a more tractable heuristic—the results in this sec-

> tion suggest two examples, where Betweenness Centrality can approximate RRTD-IDDFS and Degree Centrality can approximate RRTD-RW. While Degree Centrality is straightforward to compute, Betweenness Centrality is still computationally costly because it requires finding optimal paths for all tasks. Importantly, Betweenness Centrality has a probabilistic formulation, so it can be estimated with analytic error bounds [Borassi and Natale, 2019]. In this formulation, states that are more central appear more often in paths sampled from an appropriate distribution (i.e. sample a task, then sample an optimal path uniformly at random). This suggests a trivial memory-based strategy that tracks the occupancy of states visited along paths—when the paths are appropriately sampled, the expected occupancy should be related to Betweenness Centrality. Another approach is to approximate Betweenness Centrality, like in one planning-specific method that analyzes small regions of the environment separately, then pools this information to choose subgoals [Şimşek and Barto, 2009].

We have additionally added explicit connections to this memory-based strategy where appropriate. In describing the experimental design:

> In simulations and pilot studies, states with high visit rate coincided with the predictions of RRTD-IDDFS and Betweenness Centrality. This made it difficult to dissociate model predictions from an alternative memory-based strategy where frequently visited states are selected as subgoals. As noted in the previous section, this memory-based strategy is related to sampling-based estimation of Betweenness Centrality. To address this confound, we modified the experimental task distribution so that long trials were interleaved with *filler trials* requiring navigation to a state directly connected to the start state. These filler trials were adaptively selected to increase visits to states besides the most-frequently visited one; in pilot studies and simulations, this was sufficient to dissociate visit rate and model predictions.

And in the motivation of analyses:

> We additionally compare model predictions to participant state occupancy during navigation trials in order to assess whether

> people are relying on simple, memory-based strategies to respond to the probes, as described above.

As well as the results:

> In contrast, we found that state occupancy was a worse fit to participant behavior than either of RRTD-IDDFS or Betweenness Centrality, suggesting that the introduction of filler trials was sufficient to rule out a trivial strategy based on state occupancy.

Finally, we mention these issues in the *Discussion* (new text in bold):

> However, Betweenness Centrality is also expensive to compute since it requires finding optimal paths between all pairs of states–something our participants are not likely doing. **Since we were able to rule out one trivial estimation strategy (state occupancy) through the inclusion of filler trials, the issue of tractable estimation is an open question for future research to explore by proposing other estimation strategies and experimental manipulations to dissociate their predictions.** Identifying even more efficient approximations to resource-rational task decomposition will be essential for a process-level account of human behavior, as well as for advancing a theory of subgoal discovery for problems with larger state spaces.

> *4.) The current study presents a normative account for resource-rational behavior in graph-structured environments, where subtasks are well-defined and the state space can be decomposed with reasonable certainty, only by considering the graph structure itself (subjects transition from one state to another, transitions do not depend on skill level of executing the behavioral task at hand, or the time to complete it etc). To keep up with the general motivation of the study as put forth in the author summary and introduction ("how do people decompose tasks to begin with?", line 11), the authors could elaborate on the extent of how generalizable the presented normative accounts are to other, non-graph-structured*

planning tasks. How are sub-goals identified in the more general case, e.g. in more complex, less discrete tasks that involve more uncertainty about the state space and completion of sub-tasks/achievement of sub-goals?

We agree that broader consideration of the limits and extensibility of the framework should be added to the text. We have made a few changes in response to this comment. First, in *A Formal Framework for Task Decomposition* we note a few published extensions to an early version of framework, though all deal with problems that are discrete, deterministic, and observed:

> The formal presentation of our framework considers subgoal choice with intentionally restricted algorithms: brute-force search methods that exclude problem-specific heuristics to accelerate planning. However, the examples in this section (navigating to the post office via the café, navigating in a place with low visibility) likely rely on algorithms that incorporate heuristics, particularly related to spatial navigation. While outside the scope of this manuscript, our framework can flexibly incorporate any search algorithm that can define an algorithmic cost, including those that make use of heuristics. For example, in a previous theoretical study we applied an early version of our framework to task decomposition in the Tower of Hanoi by using A* Search [Ghallab et al., 2016] with an edit distance heuristic [Correa et al., 2020]. Our framework also considers a constrained set of task decompositions that consist of individual subgoals. In comparison, the influential *options framework* [Sutton et al., 1999] defines a more general set of hierarchical task decompositions, in which, for instance, subtasks can be defined by the subgoal of reaching any one out of a set of states and subtask completion can be non-deterministic. However, finding such subtasks remains a challenging problem in machine learning, so by focusing on the simpler problem of selecting a single subgoal we are able to make a significant amount of progress in understanding a key component of how humans plan. While not yet fully explored, our formalism can be extended to encompass broader types of hierarchy (and also varied search algorithms). For instance, another theoretical study adapted our framework to support more varied kinds of

hierarchical structure by incorporating abstract spatial subgoals in a block construction task [Binder et al., 2021].

In the *Discussion*, we note potential future extensions of our framework to model spatial navigation and settings that involve learning across tasks. We also note in passing some extensions that are further afield, like control-theoretic settings and higher-dimensional reinforcement learning settings solved through deep learning.

> As mentioned in the text, our framework and experiment explicitly focus on the constrained setting of brute–force search. However, other theoretical studies have extended this framework to incorporate heuristic search [Correa et al., 2020, Binder et al., 2021] and abstract subgoals [Binder et al., 2021]. Future research should continue to explore extensions of this framework to more robustly test the predictions of resource-rational task decomposition. For example, our framework could be used to make predictions about subgoal choice in spatial navigation tasks by incorporating spatial distance heuristics and using heuristic search algorithms like A* search or Iterative-Deepening A* search [Ghallab et al., 2016]. Another direction could explore other resource costs like memory use, motivated by the relatively low memory use of IDDFS discussed above. At present, our framework assumes planning for tasks occur independently, avoiding the reuse of previous solutions to subtasks. Despite this absence, our framework still predicts a normative benefit for problem decomposition. However, research has found that people learn hierarchically, exhibiting neural signatures consistent with those predicted by hierarchical reinforcement learning theory [Ribas-Fernandes et al., 2011]. Further research could relax the independence between task solutions by explicitly reusing solutions (as in [Solway et al., 2014]) or turning to formulations based on reinforcement learning [Harb et al., 2018], particularly those designed for a distribution of goal-directed tasks with shared structure as studied in this manuscript [Nasiriany et al., 2019]. A particularly interesting direction could incorporate model learning (as in the Dyna architecture [Sutton and Barto, 2018]) across tasks in order to explain the influence of task learning on task decomposition. Further extension of this framework could build on

resource-rational models developed in other domains, like Markov Decision Processes [Jinnai et al., 2019] and feedback control [Prystawski et al., 2022], and draw inspiration from approaches used to learn action hierarchies in high-dimensional tasks [Harb et al., 2018, Nasiriany et al., 2019].

5.) For resource-rational computations in subtask-level planning to occur, subjects would need to have access to the computational cost of a cognitive process – before deciding whether to engage in the computation, or rather not to invest time and cognitive resource. This cost is of course only available after indeed running the very same computation, which sort of seems to defeat the purpose of resource-rational deliberation. It is more of a question out of curiosity, but I assume many readers will have similar thoughts – so it might be beneficial to elaborate (e.g. in the discussion) how the necessary "ingredients" for the resource-rational deliberation are thought to be accrued before engaging in resource-rational task decomposition.

We thank the reviewer for calling attention to this. We agree that this issue should be addressed in some capacity. We have added a few sentences remark on this issue and citing a related study at the end of *Comparing Accounts of Task Decomposition*. We hope this shines some light on this broad concern that resource-rational accounts must address for feasibility.

First, finding the optimal task decomposition in a brute-force manner is more computationally expensive than simply solving the task. One alternative is to *learn* the value of task decompositions, relying on the shared structure between tasks and subgoals to ensure learning efficiency—for example, in the domain of strategy selection, one study uses shared structure to ensure efficient estimation which is incorporated by decision-theoretic methods to deal with the uncertainty in these estimates [Lieder et al. 2017].

6.) It would be beneficial to present an additional, alternative metric for model comparison that is less dependent on the assumption of uninformative (flat) priors and an approximately

multivariate Gaussian posterior distribution as the AIC. I suggest adding another metric like the WAIC, or preferably, cross-validation to assess the predictive accuracy of the models under consideration. Do other metrics produce convergent model comparison results?

We appreciate this suggestion from the reviewer and have given their feedback careful consideration. While we agree that carefully accounting for model complexity using measures like WAIC (or indeed, AIC) is critical when comparing cognitive models with different free parameters, we do not think these sophisticated measures are necessary or even appropriate in our case. To the contrary, the additional ambiguity and complexity they add, due to the proliferation of different metrics based on different construals of the bias problem and different approximations, together with different ways of applying them in the context of hierarchical models, actually obscures the straightforward question of comparative model fit here. Indeed, on reflection we believe that the invocation of AIC in our original submission was an unnecessary and potentially confusing red herring, for which we apologize.

Before explaining why, we first briefly recapitulate the analysis strategy to provide appropriate context. We predict participant choice on subgoal probes using hierarchical multinomial regression with the predictions of various theories of subgoal choice as regressors. A separate regression analysis is fit for each subgoal theory, containing a fixed and random effect for predictions of the theory. Importantly, our regressors (the predictions of the theories that are actually being compared) have no free parameters and are not fit to data; the question is just which of these structurally identical accounts provides the best fit to choices.

Because these regression analyses have identical effect structure and no theory-specific free parameters, we think that standard methods for model comparison (e.g., WAIC) are not appropriate for deciding among the theories. Instead, we feel that the question of selecting among theories is best framed by directly comparing their fit to data, e.g. by unadjusted LL. Obviously, this provides a biased estimate of predictive fit to test data (or of marginal model likelihood, on a Bayesian view) due to the free parameters in the regression model itself, which is what usually motivates the profusion of different adjusted model selection metrics in order to compare between structurally different models. But importantly, this type of overfitting contributes equivalently to each of the models here: not just the number of free parame-

ters, but the actual model structure is the same for all the competing models, with the only difference being the candidate explanatory regressor itself. In this case, adjusting for a shared bias term seems at best unnecessary, and at worst a potentially misleading source of noise. Thus, while considering this comment, we realized that even the AIC (which we used in the original draft) is just as informative as the log likelihood. The parameter penalty in AIC is equivalent for each of these regression analyses, so any comparison between them based on AIC is identical to a comparison based on the deviance or negative log likelihood. While we agree that AIC should be replaced with a more accurate approximation to predictive likelihood if it were doing useful work, we are skeptical in this case that replacing it with a more elaborate but still approximate computation would offer a more accurate view on the problem at hand.

Based on this rationale, we have decided to compare theories on the basis of the log likelihood, and have ensured that figures reflect this change, as well as noting this rationale in the text:

> Since the regression analyses have the same effect structure and the underlying theories being compared have no free parameters, we compare the relative ability of factors to predict probe choice through their log likelihood (LL) in Fig [...].

Minor questions and comments

(1) It is surprising that predictions based on betweenness centrality seem so closely aligned with predictions of RRTD-IDDFS but not with RRTD-BFS (Fig. 3), given algorithmic work suggesting that betweenness centrality can be efficiently (and probabilistically) approximated using balanced bidirectional breadth-first search (e.g. Borassi & Natale, 2019, https://doi.org/10.1145%2F3284359). The authors could clarify and discuss this.

We appreciate the reference to these recent results and the opportunity to examine this point in depth. Based on our understanding, the results in Borassi and Natale (2019) do not contradict our results, since their use of balanced bidirectional breadth-first search (bb-BFS) does not bias their

computation of betweenness centrality (BC). We include extended consideration of their results below in order to come to this conclusion. In the main text, we have added discussion of probabilistic approximation of BC when discussing tractable approximations of BC, as noted in a previous response.

Our understanding is that the general approach to probabilistic approximation of BC is based on sampling from a probabilistic definition of BC. As Borassi and Natale (2019) describe probabilistic approximation of BC $\mathrm{bc}(v)$ of a node v:

> The main idea is to define a probability distribution over the set of all paths, by choosing two uniformly random nodes s, t, and then a uniformly distributed st-path π, so that $\Pr(v \in \pi) = \mathrm{bc}(v)$. As a consequence, we can approximate $\mathrm{bc}(v)$ by sampling paths $\pi_1, \ldots, \pi_\tau$ according to this distribution, and estimating $\tilde{b}(v) := \frac{1}{\tau}\sum_{i=1}^{\tau} X_i(v)$, where $X_i(v) = 1$ if $v \in \pi_i$ (and $v \neq s, t$), 0 otherwise.

The KADABRA algorithm proposed by Borassi and Natale (2019) is a substantial speedup resulting from two contributions: an adaptive sampling scheme with error bounds, and the use of balanced bidirectional breadth-first search (bb-BFS). To the best of our knowledge, bb-BFS does not influence the adaptive sampling scheme in the text, particularly based on their adaptive sampling algorithm—which isolates the use of bb-BFS to the function `samplePath()` (see Algorithm 1)—and their proof of algorithm correctness—which does not reference bb-BFS in describing shortest paths (see proof of Theorem 4). This suggests that the use of bb-BFS primarily serves to optimize the run-time of KADABRA, but doesn't bias the results in a way that is specific to bb-BFS. This seems reinforced by the fact that the adaptive sampling scheme provides error bounds, but does not account for any bias due to the choice of search algorithm.

Further, the section describing bb-BFS is motivated in a manner consistent with this perspective: "The idea behind this technique is very simple: if we need to sample a uniformly random shortest path from s to t, instead of performing a full BFS from s until we reach t, [...]", which then goes on to describe the bb-BFS algorithm. More generally, since BC is computed without respect to a particular search algorithm and formulated assuming sampling uniformly at random from optimal paths, we think that the lack of algorithm-specific bias in KADABRA and BC does not contradict our ob-

served results, and would not lead to the prediction that BC and RRTD-BFS should make similar predictions.

> *(2) The last sentence of the abstract "Taken together, our results provide new theoretical insight into the computational principles underlying the intelligent structuring of goal-directed behavior.", seems to overstate the behavioral findings of the study and what the models represent. In my view, the study shows how well predictions of a number of considered normative accounts and heuristics are aligned with human behavior, but do not necessarily represent a proof for the use of these exact computational principles by humans (there could be alternative computational accounts and heuristics that are currently not considered in the model space of the present study – e.g. Dijkstra's algorithm for discovery of the shortest path between nodes).*

We thank the reviewer for making this point. We agree that this line should be more precisely phrased to reflect the findings in the study. We have rewritten the sentence as follows:

> Taken together, our results suggest the computational cost of planning is a key principle guiding the intelligent structuring of goal-directed behavior.

Since a key attribute of our framework is that other search algorithms can be incorporated with the definition of computational costs, we additionally make explicit reference to the extensibility of our model at the end of *A Formal Framework for Task Decomposition* and the *Discussion*, as noted in our above response to Major Question 4.

> *(3) I was a bit confused by the fact that the authors indicate the number of all possible unique 8-node graphs graph-structured planning tasks (11,117) multiple times throughout the manuscript without mentioning that this was not the number of graphs actually used in the behavioral experiment. It is more informative to learn that the authors further limited this set by ensuring that each graph had 10 distinct tasks with 3+ actions for an optimal*

solution (lines 532-533), which greatly enhances the scrutiny of the approach.

We appreciate that the reviewer made note of this. We agree that this information should be foregrounded to ensure important details of the experimental design are clearly noted. We have added explicit references to the constrained subset of 1,676 in the *Introduction*:

> To empirically evaluate this framework, we report results from a pre-registered experiment ($N = 806$) that uses 30 distinct graph-structured tasks sampled from 1,676 graphs, the subset of the 11,117 graphs that are compatible with our experimental design.

We have also mentioned this with similar language when describing the experiment in *An Empirical Test of the Framework*.

---

(4) In which way was the multiple-choice survey question at the end of the experiment used? Did it serve as an exclusion criterion? It would be beneficial to rule out the potentially confounding effects of participants using drawings or pictures of the graph and re-run the behavioral analyses only including subjects who did indeed adhere to the protocol.

We thank the reviewer for drawing attention to this potential confound. As detailed in the results, we have added a supplementary analysis predicting subgoal choice, restricted to the subset of participants that did not draw a map. We found results that were consistent with those in our initial submission.

> Since the experiment was designed to so that only local connections were visible during navigation trials but conducted via an online platform, in the closing survey we asked participants if they used a reference to the task structure besides the interface ("Did you draw or take a picture of the map? If you did, how often did you look at it?"). Participant responses were as follows: 603 participants selected "Did not draw/take picture", 65 selected "Rarely looked", 90 selected "Sometimes looked", and

48 selected "Often looked". In order to ensure the above results were not impacted, we ran the same analysis in the subset of participants ($N = 603$) that selected "Did not draw/take picture" and found qualitatively similar results (in Appendix).

The below figure was included to support these results.

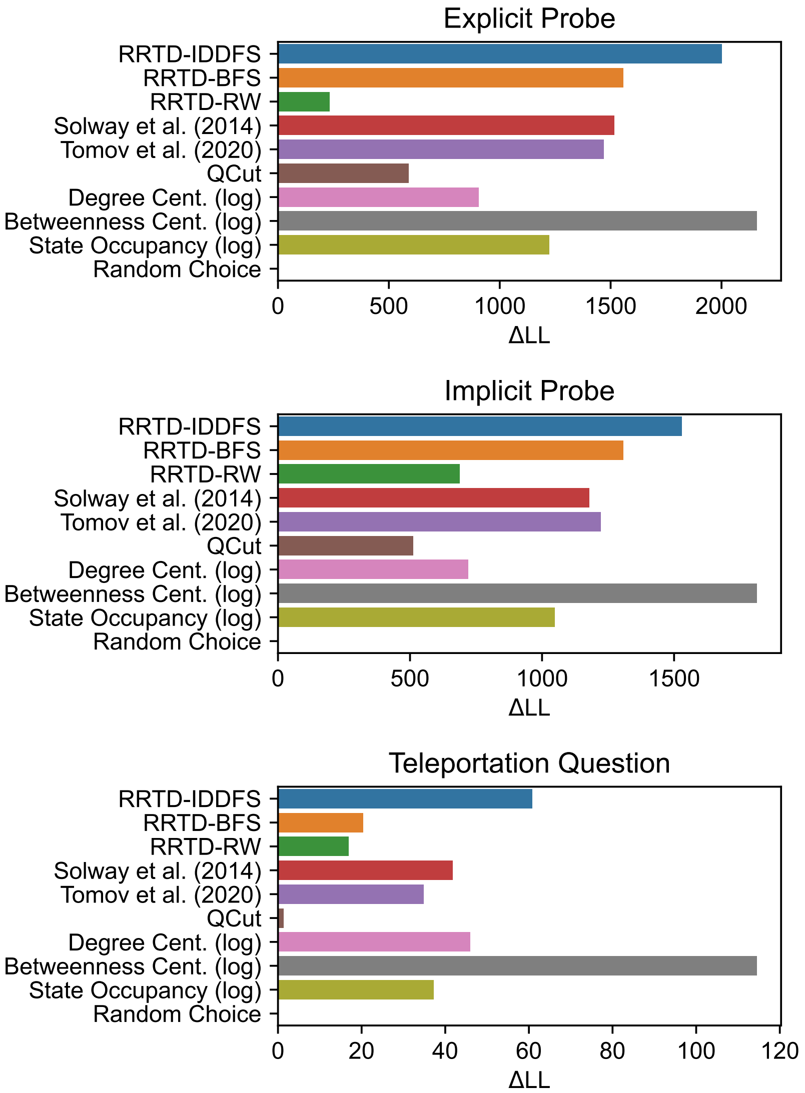

Caption: Comparison of probe choice behavior in the subset of participants ($N = 603$) that reported not using an additional

visual aid during the experiment. Analysis is otherwise identical as that in main text, using mixed-effects multinomial regression to predict subgoal choice behavior for each subgoal probe. Log likelihood (LL) is relative to the minimum model LL for each probe. Larger values indicate better predictivity.

---

(5) The explanations of the toy example task decomposition in the figure caption of Fig. 1 (c-e, page 3) are a bit unclear without reading the section describing how the task was set up in the main manuscript (only at page 6). Please expand the figure caption such that this becomes clearer without having to refer to the main text.

Based on this comment, we have split the figure so that the toy example is closer to the text where it is referenced. We have also added additional context to the figure, in the hopes that it will be easier to understand without needing to reference the text. For the purpose of the example in this reviewer response, we only include the search costs for IDDFS.

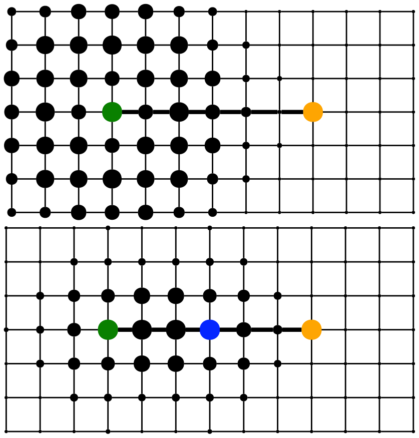

Caption: State-specific search costs of the algorithms. The depicted task requires navigating on a grid from the start state (green) to the goal state (orange) with fewest steps. Each column corresponds to a different algorithm and demonstrates two scenarios—*Top*: Search cost without subgoals, *Bottom*: Search cost when using the path midpoint as a subgoal (blue). We define the search cost as the number of iterations required for the

search algorithm to find a solution. Larger states were considered more often during the search algorithm, resulting in greater search cost.

---

(6) Page 12, lines 354-355: I do not understand the pre-registered exclusion criterion of "no more than 175% of the optimal number of actions". Is this a typo?

We have rewritten this exclusion criteria to emphasize that we are eliminating participants with inefficient behavior:

> Of the 952 participants that completed the experiment, 806 (85%) satisfied the pre-registered exclusion criteria requiring efficient performance on the navigation trials. If a participant took 75% more actions than the optimal path (averaged across the last half of "long trials"), their data was excluded.

---

(7) Page 13, line 403: The last sentence before Figure 5 seems to overstate the behavioral findings. I do not think that the presented analyses (of internal consistency) are sufficient to establish validity of the construct (from a test theoretic perspective) – internal consistency is a metric of reliability. Please rephrase this.

We thank the reviewer for this feedback, and agree that the language used to conclude this section should be more precise to ensure it accurately reflects the results:

> In sum, these results suggest the subgoal probes are well-correlated, though to a lesser degree for the Teleportation Question. They also suggest a strong connection between the probes and planning behavior.

> *(8) Figure 3: Why are there relatively low correlations between RRTD-RW and Q-Cut model predictions, if one is a heuristic approximation of a random walk, while correlations e.g. between RRTD-IDDFS and betweenness centrality are much higher. Was another than rank-one approximation used?*

We thank the reviewer for the careful reading of these analyses. In the appendix, we included a discussion about the relationship between QCut and RRTD-RW at varying degrees of approximation. In brief, increasing the rank of the approximation is sufficient to decrease the correlation between QCut and RRTD-RW. In this updated version of the manuscript, we have added an explicit note for the case where the simulations should have perfect alignment with our formal analysis—in particular, rank-one RRTD-RW has perfect correlation with QCut for regular graphs:

> Our formal analysis makes a stronger prediction for the case of regular graphs—in particular, QCut should be completely correlated with a rank-one approximation of RRTD-RW. When restricting attention to regular graphs, we find that QCut and rank-one RRTD-RW have a correlation of one, aligning with the formal analysis.

In the course of verifying this quantity, we further clarified how the low-rank approximation is computed in the Appendix. In addition, we found a discrepancy with the figure relating varying degrees of approximation of RRTD-RW to QCut and Degree Centrality. In the original submission, an incorrect task distribution was used. Only the left panel of this figure was impacted and has been updated to use the correct task distribution in the updated manuscript. While there are small quantitative changes, the results are still consistent with the reported qualitative results, where QCut is most related to RRTD-RW for a rank 1 approximation, but for larger ranks, Degree Centrality is more related to RRTD-RW. Notably, the correlations for full-rank RRTD-RW are now the same quantities reported in the correlation analysis between models. We apologize for this error. The updated figure and original caption are below.

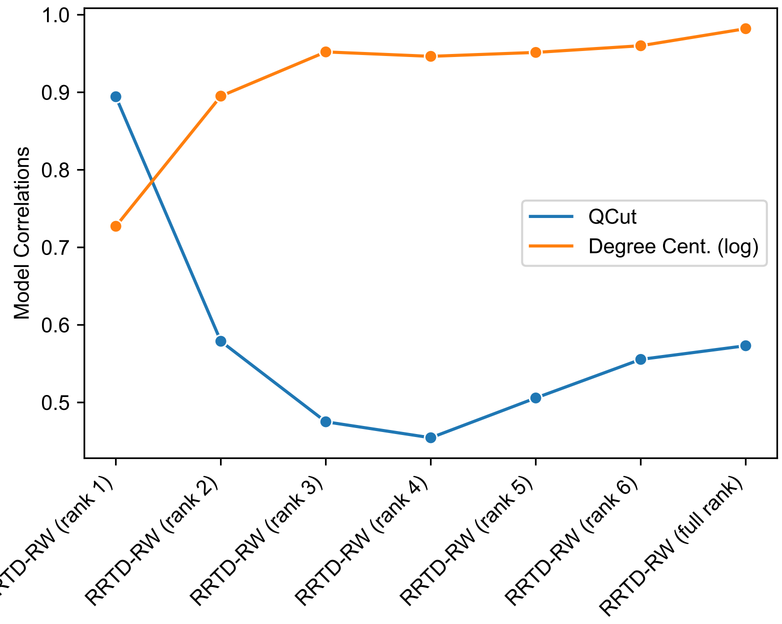

Caption: Correlation of two models (QCut in blue and Degree Centrality in orange) plotted as a function of rank of a spectral approximation to RRTD-RW using the below formula for rank-k' spectral approximation. Correlations were computed as in Fig 4.

---

(9) The Github link to data and code used for analysis (https://github.com/cgc/resource-rational-task-decomposition) is currently not working, please make this important information available.

We are deeply sorry for this error on our part. In our initial submission, we attached a private link (https://anonymous.4open.science/r/resource-rational-task-decomposition-0CED) for data and code review as a "Data Review URL" in the "File Inventory". Since we only intended this private link to be accessible during review, we had links in the text point to the link where the code would eventually be publicly accessible. In order to avoid any further confusion in the review process, the code is publicly accessible at https://github.com/cgc/resource-rational-task-decomposition and all links in the text have been updated.

Reviewer 3

We are very grateful to the reviewer for carefully reading of our manuscript and providing helpful comments and perspectives. Based on this feedback, we have added additional discussion and analyses, which we hope will serve to address the reviewer's concerns.

Below, the reviewer's original comments are in italics and our responses proceed in non-italics. Quotes from the updated manuscript are in blue.

> *In summary, my view is that this is a well-executed study which makes a significant contribution to the literature on human planning. In particular, the authors are to be commended on their efforts to integrate a variety of hitherto disparate studies within a unified perspective under the framework of resource-rational DM. Furthermore, a more detailed analysis of the hierarchy/bottleneck problem is presented based on the most comprehensive experiment on this topic to date. However, there are a couple of important gaps in the data analysis approach in my view.*
>
> *Regarding the data analysis and model comparisons. The authors emphasize the algorithm-based approach in contrast to the structure inference approach. I agree with this perspective and find it interesting however it seems to me that this suggests an investigation into what planning algorithm is being used by the participants. To put it bluntly, what is the utility of considering a model such as RRTD-IDDFS to predict subgoals if the participants are not using IDDFS to plan? I wonder if the authors could at least provide some perspectives on this if not actually run some model fits/comparisons on choices during the navigation trials.*

We appreciate the reviewer's directness about this issue. It is an issue that we discussed while initially writing the manuscript, so we do think that it is particularly important.

We think that the present experimental design makes it difficult to identify signatures of search algorithms. Our experiment is not designed for process tracing, so the data from our experiment can not be explicitly compared to the computations in a search algorithm. In addition, our experience is that the probabilities that optimal search algorithms (like BFS or IDDFS)

assign to paths tend to vary with extreme subtlety. For that reason, we also think it would be difficult to compare choices among paths to those predicted by different search algorithms. We have incorporated mention of these issues when discussing experimental limitations in the *Discussion*:

> A third limitation is that the navigation trials provide limited insight into the algorithms people are using to plan. While participants only see local connections during navigation, analogous to the local visibility search algorithms have, there are a number of reasons why their navigation behavior would be difficult to relate to the choice of search algorithm. The main issue is that planning steps are generally covert, and do not correspond in any simple way with steps of overt behavior given by the plan that is ultimately produced. Such covert planning is better suited, in future work, to being studied through process-tracing experiments [Callaway et al., 2022, Ho et al., 2022], think-aloud protocols, or by investigating neural signatures related to planning and learning [Liu et al., 2021]. It is possible that some aspects of planning are externalized in the current experiment, via exploration on navigation trials, but this is at best incomplete. For instance, participant behavior improves over the course of navigation trials, suggesting they are performing mental search instead of search via navigation. Also, the experiment restricts single-step movement to neighboring states, whereas by contrast, algorithms like BFS might plan over states in an order where subsequent states are not neighbors. Identifying the search algorithm participants use is critical for future studies, since our framework predicts that task decomposition is driven by the search algorithm used.

We do feel that this issue is critical, and hope that our findings can justify further study of the search algorithms people use. We note in the *Discussion* that we hope future research may shed light on these issues through process-tracing paradigms or neuroscientific methods.

> *Related to this, it seems that an immediate computational hypothesis emerging from the normative framework studied here (and the principle of subgoaling more generally) regards the modulation of reaction times. That is, given a task decomposition, then*

> *subtask-level planning should occur at a subgoal specifically and this should be reflected in reaction times. More generally, reaction times can be an important behavioural indicator of internal computation and I think it should be somehow addressed in this study.*

We thank the reviewer for suggesting this, since our initial submission had little analysis of participant response times. We have developed an additional analysis to address this issue, which has been added to the appendix in *Predicting navigation response times*. In the analysis, we find that participant response times are slower at their self-reported subgoals when examining navigation trials that can be matched to probe trials. We also perform a separate analysis using subgoal predictions from the various theories to predict response times during navigation. We find affirmative evidence that is largely consistent with results in the remainder of the manuscript.

> We first examined how participant response times vary during navigation, focusing on the difference between response times at self-reported subgoals and other states. For simplicity, we examine navigation trials with optimal behavior and an associated Explicit Probe trial where participants reported a subgoal that they also visited during navigation. First, we found that participants respond most slowly at the initial state ($M = 5.36s$, $SD = 7.58$), when compared to their responses at their self-reported subgoals ($M = 2.00s$, $SD = 1.38$; $t(789.8) = 11.9$, $p < .001$) or other states ($M = 1.70s$, $SD = 1.12$; $t(773.4) = 13.0$, $p < .001$). We also found that participants respond more slowly at their self-reported subgoal than at other states ($t(1423.9) = 4.6$, $p < .001$). We found similar results for the Implicit Probe trials. These findings could indicate that participants defer making detailed plans until they are necessary—that is, after reaching a subgoal, participants figure out how to reach the subsequent subgoal or goal.
>
> Based on this result, we used model predictions for subgoals to predict response times during navigation. We analyzed every state choice during long navigation trials where participants took an optimal path. In order to focus on response times at subgoals compared to other states, we excluded the initial state to avoid any effect from initial planning. We found results that were

largely qualitatively consistent with those observed elsewhere in the study (Figure inline and Table in manuscript):

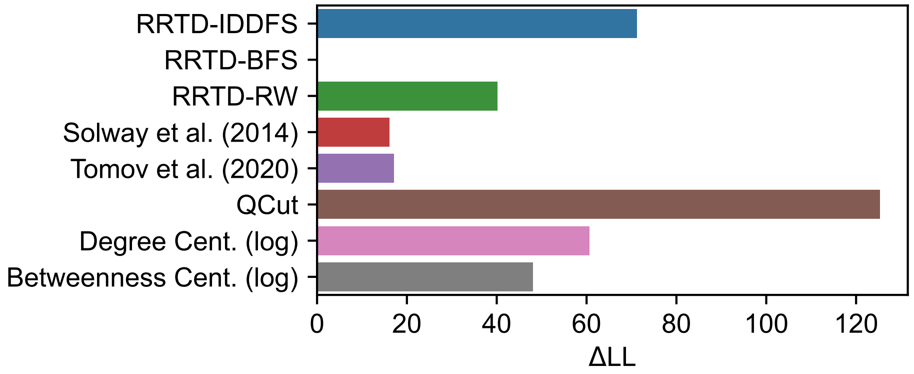

Caption: Predicting navigation response times using hierarchical linear regression. Log likelihood (LL) is relative to the minimum model LL. Larger values indicate better predictivity.

The models that fit the observed data best included Betweenness Centrality and RRTD-IDDFS. Most models had positive coefficients, consistent with the idea that response times should be higher at states that are more likely to be subgoals. However, two other models also fit the data well: Degree Centrality and QCut. The efficacy of Degree Centrality as a predictor suggests that people simply take longer to act at states that have greater degree (i.e. states that have more edges). The efficacy of QCut is more difficult to interpret in the context of our other results, particularly since it has a negative coefficient and is the best fit to the data.

Hierarchical linear regression analyses were fit using `lme4` and predicted the logarithm of participant response times in seconds. The regression models included an intercept and slope for subgoal predictions as effects at three levels: fixed effects, random effects for each graph, and random effects for each participant. In addition, they included a fixed effect of the trial number, in order to fit differences resulting from learning over the course of trials.

> Because the regression models have identical effect structure, we compare them by log likelihood (LL).

We do note that these were posthoc analyses and the experiment was not designed to assess response times in the *Discussion*:

> Second, though we report analyses of participant response times, these analyses were not pre-registered and our experiment was not designed to assess response times. These findings should be reevaluated in experimental designs appropriate to assess response times. For example, we found that participants were slower to respond at their subgoals, suggesting they were planning at those states. However, our normative framework makes no prediction about *when* planning occurs. The framework only defines a resource-rational value for subgoals that can be used to simplify planning whether it happens before action or after reaching a subgoal. Future experiments could investigate this further through manipulations to influence when planning is employed, like a timed phase for up-front planning or incentive for fast plan execution.

> *I think the authors could tune their introduction to the literature a bit better. For example, on the critical idea of relating task decompositions to planning (rather than structure inference), it is said that "...our framework differs from many existing accounts because we directly incorporate planning costs into the criteria used to choose a task decomposition." I think it should be acknowledged that this idea is not fundamentally new and existing accounts have already considered planning costs in task decompositions computationally e.g. Jinnai et al 2019 (in RL) and McNamee et al 2016 (regarding human planning) (both cited here but there may be others). In particular, the latter considers a random walk search policy and points to log(degree centrality) as a key variable in determining decompositions/subgoals consistent with the modelling results here (see Fig 3 RRTD-RW vs degree centrality (log)). I think the specific computational novelty here is the integrative framework (which generates new results).*

We thank reviewer for this feedback, and agree that more explicit coverage of the relationship between our framework and previous research is necessary. In the *Introduction*, we introduce the model as a direct extension of previous accounts that account for planning costs when choosing task decompositions:

> Instead, our formal framework extends research that performs task decomposition based on algorithm-specific planning costs—some algorithms previously studied are value iteration [Jinnai et al. 2019], random walk search [McNamee et al. 2016], and random sampling of optimal behavior [Solway et al. 2014]. Generalizing beyond a fixed algorithm, our framework explicitly considers how planning efficiency shapes hierarchical representations, which we use to demonstrate how resource-rational task decompositions change with varied search algorithms.

In addition, in *Comparing Accounts of Task Decomposition* we have made sure to reference the result from McNamee et al. 2016 that was noted by the reviewer. We are particularly grateful to the reviewer for letting us know about this connection.

> This relationship between RRTD-RW and Degree Centrality is qualitatively consistent with a published result that relates Degree Centrality to the task decomposition that minimizes a search cost related to RW [McNamee et al. 2016].

> *Minor comment:*
>
> *Can authors speculate on the low consistency of teleportation probe? As I understand it, this measure is taken once per subject at the end of the experiment thus I would intuitively expect this measure of subgoals to be stable as opposed to the other measures which may be varying throughout the experiment.*

We thank the reviewer for calling our attention to this issue. We think that the relatively low consistency of a teleportation probe directly results from the smaller number of samples (one) that it has, compared to the relatively large number of samples of the other probes (10). This makes it

difficult to compare the self-consistency of these different probe types. In order to avoid an invalid interpretation, we explicitly describe this issue in the text:

> While the Teleportation Question exhibits relatively low self-consistency and cross-probe consistency, it is difficult to compare to the consistency of the other probes since both the Explicit and Implicit probes were sampled for 10 different tasks per participant, while the Teleportation Question was only sampled once per participant.
